# Multiple Mechanisms for Copper Uptake by Methylosinus trichosporium OB3b in the Presence of Heterologous Methanobactin

**DOI:** 10.1128/mbio.02239-22

**Published:** 2022-09-21

**Authors:** Peng Peng, Wenyu Gu, Alan A. DiSpirito, Jeremy D. Semrau

**Affiliations:** a Department of Civil and Environmental Engineering, University of Michigan, Ann Arbor, Michigan, USA; b Roy J. Carver Department of Biochemistry, Biophysics and Molecular Biology, Iowa State University, Ames, Iowa, USA; University of Arizona

**Keywords:** TonB-dependent transporter, copper, methanobactin, methanotroph

## Abstract

Methanotrophs require copper for their activity as it plays a critical role in the oxidation of methane to methanol. To sequester copper, some methanotrophs secrete a copper-binding compound termed methanobactin (MB). MB, after binding copper, is reinternalized via a specific outer membrane TonB-dependent transporter (TBDT). Methylosinus trichosporium OB3b has two such TBDTs (MbnT1 and MbnT2) that enable M. trichosporium OB3b to take up not only its own MB (MB-OB3b) but also heterologous MB produced from other methanotrophs, e.g., MB of Methylocystis sp. strain SB2 (MB-SB2). Here, we show that uptake of copper in the presence of heterologous MB-SB2 can either be achieved by initiating transcription of *mbnT2* or by using its own MB-OB3b to extract copper from MB-SB2. Transcription of *mbnT2* is mediated by the N-terminal signaling domain of MbnT2 together with an extracytoplasmic function sigma factor and an anti-sigma factor encoded by *mbnI2* and *mbnR2*, respectively. Deletion of *mbnI2R2* or excision of the N-terminal region of MbnT2 abolished induction of *mbnT2*. However, copper uptake from MB-SB2 was still observed in M. trichosporium OB3b mutants that were defective in MbnT2 induction/function, suggesting another mechanism for uptake copper-loaded MB-SB2. Additional deletion of MB-OB3b synthesis genes in the M. trichosporium OB3b mutants defective in MbnT2 induction/function disrupted their ability to take up copper in the presence of MB-SB2, indicating a role of MB-OB3b in copper extraction from MB-SB2.

## INTRODUCTION

Methanotrophs, microbes that use methane as their sole carbon and energy source (1–3), have received increased attention as they play a critical role in controlling emissions of methane, a potent greenhouse gas with a global warming potential 28 times higher than carbon dioxide over a 100-year time frame (4). Interestingly, methanotrophic activity is strongly controlled by copper. More specifically, copper availability controls the expression and activity of alternative forms of the methane monooxygenase (MMO) that carries out the first step in methane oxidation (i.e., the conversion of methane to methanol) (5–9). There are two forms of MMO: the cytoplasmic or soluble methane monooxygenase (sMMO) and the membrane-bound or particulate methane monooxygenase (pMMO). Most methanotrophs can only express pMMO (1, 2), and its activity is strongly dependent on copper (5–9). A small number of methanotrophs can also express sMMO in addition to pMMO (10), and in these methanotrophs, the expression and activity of the two forms of MMO are dependent on copper availability. That is, there is a “copper switch” in which sMMO expression/activity is only observed in the absence of copper, while pMMO expression/activity increases with increasing copper (11–13). In addition to controlling MMO expression and activity, copper also controls the formation of intracytoplasmic membrane of methanotrophic cells, as well as expression of genes involved in copper uptake (14, 15).

Due to the essential role of copper in methanotrophic physiology, some methanotrophs secrete a copper-binding compound or chalkophore named methanobactin (MB) for copper uptake. MBs are small (<1,350 Da) ribosomally synthesized posttranslationally modified polypeptides (RiPPS) that have high affinity and specificity for copper (~10^20^ to 10^30^ M^−1^) (2, 16–25). The copper ligands of MB consist of nitrogen-containing heterocyclic rings and neighboring thioamide groups with posttranslational modifications (2, 16–20).

Methylosinus trichosporium OB3b is a model methanotrophic type strain that was first isolated and described in 1970 (26). The genome of M. trichosporium OB3b encodes both pMMO (encoded by *pmo*CAB or *pmo* operon) and sMMO (encoded by *mmoXYBZDC* or *mmo* operon) (27), and their expression and activity is controlled by copper availability (12). M. trichosporium OB3b can produce MB to sequester copper from its habitat environment. The responsible gene cluster (*mbn*) for MB biosynthesis includes *mbnA*, encoding the MB polypeptide precursor (*mbnA*), as well as several genes either with experimentally determined roles (e.g., *mbnBC* and *N*, involved in formation of heterocyclic rings) or imputed from bioinformatic analyses (e.g., *mbnM*, believed to be responsible for MB secretion), as well as some genes with as-yet-unknown roles (e.g., *mbnPH*, encoding a diheme cytochrome *c* oxidase and its partner protein) (19, 28–30).

Structural and bioinformatic analyses indicate that MB can be divided into two general groups: group I and II. Group I MBs are typically represented by MB from M. trichosporium OB3b (MB-OB3b). The primary structure of MB-OB3b includes two oxazolone rings with a disulfide bridge between two cysteine residues (Fig. S1A). Copper is chelated by the N- and S-ligands of the oxazolones and thioamides, respectively, forming a pyramid-like shape (17, 18, 31). Group II MBs are represented by the MB from Methylocystis sp. strain SB2 (MB-SB2). Unlike MB-OB3b, MB-SB2 has one oxazolone ring and one imidazolone ring (Fig. S1B). A disulfide bridge is absent from MB-SB2. MB-SB2 forms a hairpin shape upon binding copper via the N-ligand of the oxazolone/imidazolone and the S-ligand of thioamides (17, 21). Interestingly, recent phylogenetic analyses suggest that these two general groups can be further subdivided into at least five subgroups: groups IA, IB, IIA, IIB, and IIC (28). To date, 22 *mbn* gene clusters have been identified in methanotrophs belonging to the genera Methylosinus and Methylocystis; 10 of these encode group I MB (group IA: 6, group IB: 4), and 12 encode group II MB (group IIA: 6, group IIB: 5, group IIC: 1) (28).

10.1128/mbio.02239-22.3 FIG S1 Primary structures of methanobactin from M. trichosporium OB3b (A) and Methylocystis sp. strain SB2 (B). Download FIG S1, DOCX file, 0.3 MB. Copyright © 2022 Peng et al. 2022 Peng et al. https://creativecommons.org/licenses/by/4.0/ This content is distributed under the terms of the Creative Commons Attribution 4.0 International license.

It should be stressed that MBs are part of an extracellular mechanism for copper collection; i.e., after biosynthesis, MB must be secreted and subsequently reinternalized after binding copper. The mechanism of MB excretion is believed (but not conclusively shown) to occur via a multidrug export pump encoded by *mbnM*. MB uptake has been much more extensively characterized, with a TonB-dependent transporter (TBDT, encoded by *mbnT*) shown to be critical for reinternalization (32–34). In general, TBDTs consist of two conserved domains: one a barrel domain that forms the basic/main structure and the other a plug domain that is folded into the barrel interior (35). The plug domain functions to bind and transport specific compounds, such as MB, siderophores, vitamins, nickel complexes, and carbohydrates (35, 36). Some TBDTs also include a signaling domain at the N terminus that is involved in signal transmission to regulators (i.e., extracytoplasmic function [ECF] sigma and anti-sigma factors) for inducing specific gene(s) expression (e.g., TBDT genes) (36–39). Such TBDT signaling domain and (anti)-sigma factor-mediated regulation of gene(s) expression has been extensively studied in microbial ferric-siderophore uptake systems. It has been shown that upon binding of a specific siderophore to its TBDT transporter, a conformational change of the TBDT occurs that generates a signal that is transmitted to the anti-sigma factor via the signaling domain of the TBDT, leading to release of the ECF sigma factor into the cytoplasm. The released ECF sigma factor then enables binding of RNA polymerase to the promoter of specific gene(s), thereby initiating transcription (35, 37, 40).

Previously, it has been demonstrated that M. trichosporium OB3b has two MB uptake systems mediated by MbnT1 and MbnT2 responsible for uptake of homologous and heterologous MBs (MB-OB3b and MB-SB2), respectively (32–34). In this study, we demonstrate that M. trichosporium OB3b can take up copper from heterologous MB-SB2 via two different approaches. One is initiating transcription of MbnT2, which is collaboratively mediated by the N-terminal signaling domain of MbnT2 together with an ECF sigma and an anti-sigma factor (MbnI2 and MbnR2). The other approach is using its own MB-OB3b to extract copper from MB-SB2.

## RESULTS

### Comparison of the *mbnT2* and *mbnT* gene clusters in M. trichosporium OB3b and Methylocystis sp. SB2, respectively.

**FIG 1:**
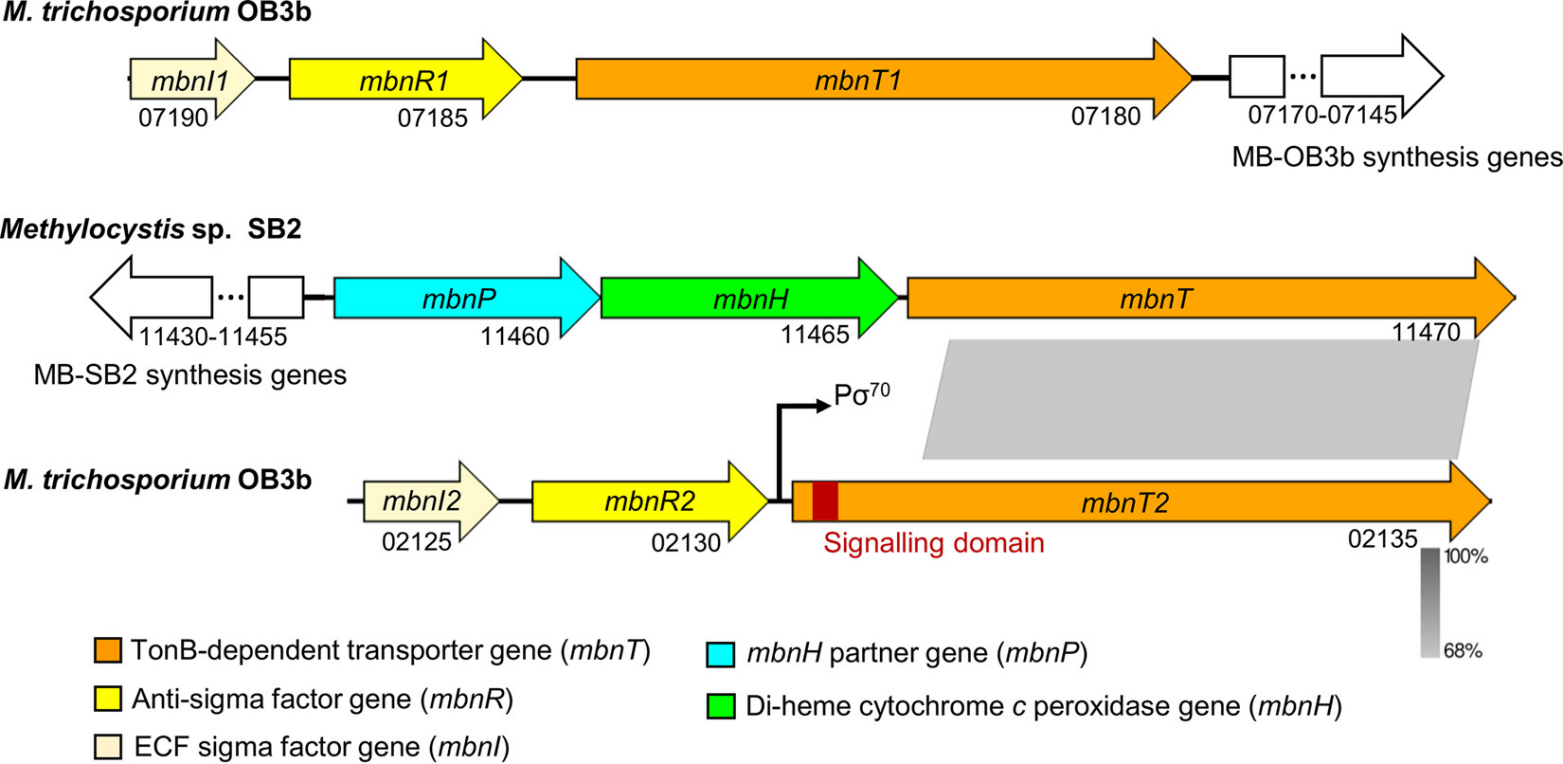
Comparison of the *mbnT* gene clusters of M. trichosporium OB3b and Methylocystis sp. SB2. Gene locus tags according to the genomes of M. trichosporium OB3b and Methylocystis sp. SB2 in NCBI under accession numbers NZ_CP023737 and NZ_CP091318, respectively, are indicated. The signaling domain encoding region of *mbnT2* (M. trichosporium OB3b) and the sequence identity of *mbnT2* and *mbnT* (Methylocystis sp. SB2) are indicated. The σ^70^ promoter (Pσ^70^) region is indicated in the *mbnT2* gene clusters. The σ^70^ promoter was predicted using BPROM. The linear comparison figure was created using Easyfig (58). ECF, extracytoplasmic function.

To visualize the similarities and differences between the MB-SB2 uptake systems in M. trichosporium OB3b and Methylocystis sp. SB2, we first compared the corresponding *mbnT* gene clusters. The nucleic acid sequences of *mbnT2* and *mbnT* of Methylocystis sp. SB2 (*mbnT*-SB2) are 68% identical (Fig. 1). The main difference between *mbnT2* and *mbnT*-SB2 was found in the initial 5′ region. That is, *mbnT2* has an extension that is missing in *mbnT*-SB2 (Fig. 1). We speculated this extension region encodes a signaling domain involved in regulating expression of *mbnT2*. Indeed, two genes encoding an ECF sigma factor (*mbnI2*) and a putative membrane sensor (anti-sigma factor, *mbnR2*) that are commonly associated with TBDTs are immediately upstream of *mbnT2* (Fig. 1). In comparison, no such regulatory genes are colocated with *mbnT*-SB2 in the genome of Methylocystis sp. SB2. Rather, upstream of *mbnT*-SB2 are *mbnPH*. As noted above, these genes encode a diheme cytochrome *c* peroxidase and its partner protein and are not believed to have any regulatory function but are speculated to assist in MB maturation and/or facilitate copper release from MB (41) (Fig. 1).

**FIG 2:**
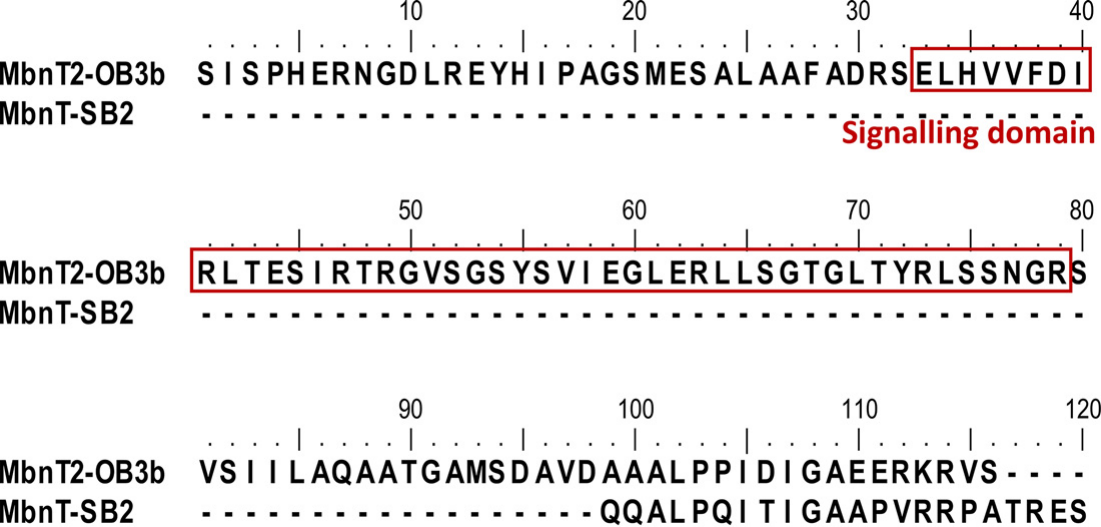
N-terminal extension (signaling domain) of mature MbnT2 protein of M. trichosporium OB3b compared to mature MbnT protein of Methylocystis sp. SB2. The signaling domain of MbnT2 was searched and predicted using Pfam database (59). The whole sequence alignment of different functional domains of MbnT2-OB3b and MbnT-SB2 are provided in the supplemental materials.

The protein sequences of MbnT2 and MbnT-SB2 both contain leading signal peptides that are responsible for translocating the proteins to the outer cellular membrane (Fig. S2). Mature protein sequence of MbnT2 and MbnT-SB2 is obtained by removing the leading signal peptides from the original protein sequences (Fig. S3), with the overall amino acid identity of mature MbnT2 and MbnT-SB2 being 57%. Sequence alignment further indicated the presence and absence of the N-terminal signaling domain in MbnT2 and MbnT-SB2, respectively (Fig. 2; Fig. S3). The amino acid identity of the conserved domains—i.e., the “plug” (responsible for binding and transport of MB) and the “barrel” (responsible for forming the basic/main structure of MbnT2)—of MbnT2 and MbnT-SB2 were 69 and 61%, respectively.

10.1128/mbio.02239-22.4 FIG S2 Signal peptide (SP) and cleavage site (CS) prediction for the MbnT2 of M. trichosporium OB3b and MbnT of Methylocystis sp. SB2 using SignalP-6.0 that can predict all known types of SPs based on protein language models (Teufel F, Almagro Armenteros JJ, Johansen AR, Gíslason MH, Pihl SI, Tsirigos KD, Winther O, Brunak S, von Heijne G, Nielsen H. 2022. SignalP 6.0 predicts all five types of signal peptides using protein language models. Nat Biotechnol 40:1023–1025). The N-terminal region, hydrophobic region, and C-terminal region of the signal peptide are labeled as “N” (marked in red), “H” (marked in orange), and “C” (marked in yellow), respectively. The CS site is indicated with a green dashed line. Download FIG S2, DOCX file, 0.3 MB. Copyright © 2022 Peng et al. 2022 Peng et al. https://creativecommons.org/licenses/by/4.0/ This content is distributed under the terms of the Creative Commons Attribution 4.0 International license.

10.1128/mbio.02239-22.5 FIG S3 T-Coffee multiple sequence alignment (Notredame C, Higgins DG, Heringa J. 2000. T-Coffee: a novel method for fast and accurate multiple sequence alignment. J Mol Biol 302:205–217) of mature MbnT2 of M. trichosporium OB3b and MbnT of Methylocystis sp. SB2. The signal peptide sequences (shown in Fig. S2) were removed from the original MbnT2 and MbnT sequences of M. trichosporium OB3b and Methylocystis sp. SB2, respectively. White letters on a black background indicate amino acids that are identical in the sequences. The protein domains of MbnT2-OB3b and MbnT-SB2 were searched and predicted using Pfam database. Download FIG S3, DOCX file, 0.1 MB. Copyright © 2022 Peng et al. 2022 Peng et al. https://creativecommons.org/licenses/by/4.0/ This content is distributed under the terms of the Creative Commons Attribution 4.0 International license.

### Copper uptake, MbnT2 and MMOs expression in M. trichosporium Δ*mbnI2R2* and Δ*mbnT2*-signal domain mutants.

To explore the function and interaction of MbnI2R2 and the signaling domain of MbnT2, we constructed two mutants in M. trichosporium OB3b in which either *mbnI2R2* or the signaling domain encoding region of *mbnT2* was deleted (Fig. S4). The absence of *mbnI2R2* and the *mbnT2*-signaling domain in the constructed mutants was confirmed by PCR (Fig. S5) and sequencing (data not shown).

10.1128/mbio.02239-22.6 FIG S4 Schematic representation of the construction of M. trichosporium mutants used in this study. Download FIG S4, DOCX file, 0.1 MB. Copyright © 2022 Peng et al. 2022 Peng et al. https://creativecommons.org/licenses/by/4.0/ This content is distributed under the terms of the Creative Commons Attribution 4.0 International license.

10.1128/mbio.02239-22.7 FIG S5 Verification of the targeted gene deletion in the constructed M. trichosporium mutants by PCR with genomic DNAs extracted from wild-type M. trichosporium OB3b and the mutants. The PCR regions for verification are indicated. The deletion region of each gene is marked (in gray) in the *mbnT2* gene cluster. The *mbnA* gene was also used for verification of the double mutants. The *mbnT1* gene was used as a control for verification of the Δ*mbnAN* Δ*mbnT2* mutant. Download FIG S5, DOCX file, 0.3 MB. Copyright © 2022 Peng et al. 2022 Peng et al. https://creativecommons.org/licenses/by/4.0/ This content is distributed under the terms of the Creative Commons Attribution 4.0 International license.

**FIG 3:**
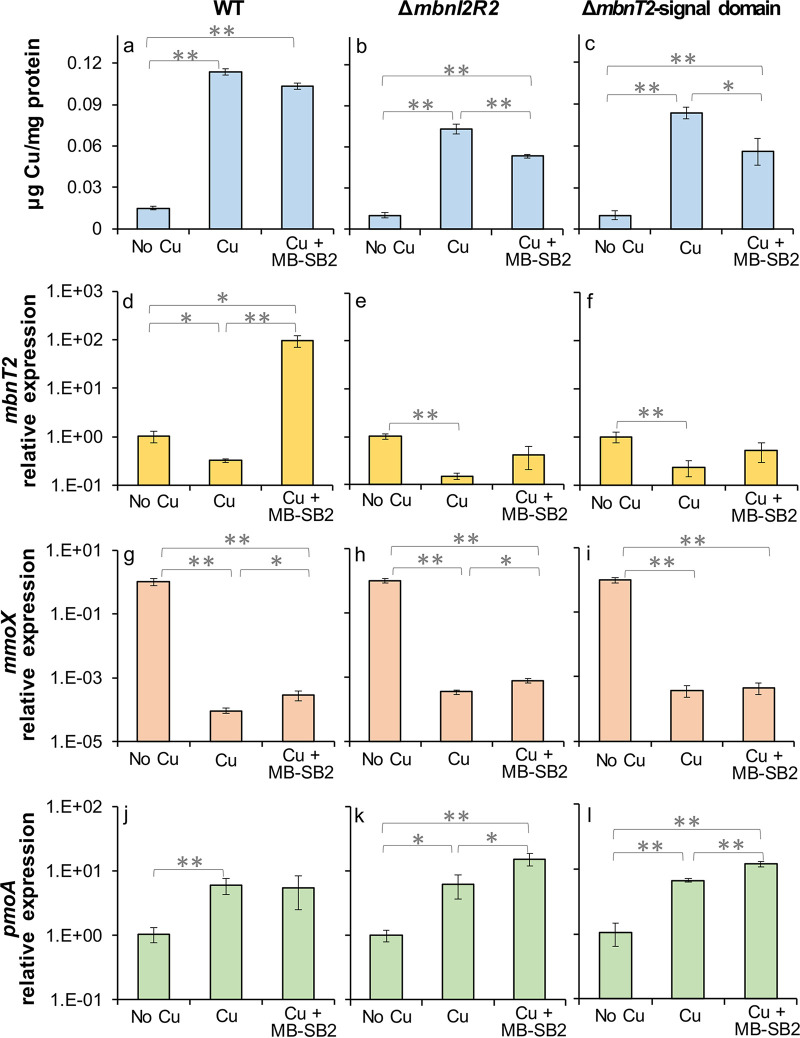
Copper associated with biomass and reverse transcription-quantitative PCR (RT-qPCR) analysis of the relative expression of *mbnT2*, *mmoX*, and *pmoA* in M. trichosporium OB3b wild type (WT) (a, d, g, and j) and Δ*mbnI2R2* (b, e, h, and k) and Δ*mbnT2*-signal domain (c, f, i, and l) mutants growing with no added Cu, 1 μM Cu, or 1 μM Cu + 5 μM MB-SB2. Error bars indicate standard deviations from triplicate biological cultures. *t* tests were performed for variance analysis between different growth conditions. *, 0.01 < *P* < 0.05; **, *P* < 0.01.

Copper associated with biomass was 7- to 8.5-fold higher in M. trichosporium OB3b wild type, Δ*mbnI2R2* and Δ*mbnT2*-signal domain mutants grown with copper (1 μM) versus without copper (*P* < 0.01; Fig. 3a to c). In the presence of 1 μM copper + 5 μM MB-SB2, copper associated with biomass in these two mutants was ~20% lower than when grown in the presence of copper (1 μM) alone (Fig. 3b and c). Such a difference in copper uptake by the mutants in the presence of copper (1 μM) alone versus copper (1 μM) + MB-SB2 (5 μM) was significant (*P* < 0.05) and was not observed in wild-type M. trichosporium OB3b (Fig. 3a).

*mbnT2* was significantly downregulated in the presence versus the absence of copper (~3-fold; *P* < 0.05) in M. trichosporium wild type. *mbnT2*, however, was upregulated (90- to 290-fold; *P* < 0.01) in M. trichosporium OB3b wild type when grown in the presence of copper (1 μM) and MB-SB2 (5 μM) compared to the absence or presence of copper (34) (Fig. 3d). Similar to that found for wild-type M. trichosporium OB3b, the presence of copper reduced *mbnT2* expression in both M. trichosporium Δ*mbnI2R2* and Δ*mbnT2*-signal domain mutants (7 and 5-fold, *P* < 0.01). Unlike wild-type M. trichosporium OB3b, however, there was no significant difference in *mbnT2* expression in both M. trichosporium Δ*mbnI2R2* and Δ*mbnT2*-signal domain mutants grown with copper (1 μM) alone versus copper (1 μM) + MB-SB2 (5 μM) (Fig. 3d to f). Moreover, there was no significant difference in *mbnT* expression in Methylocystis sp. SB2 grown in the presence of copper (1 μM) alone versus copper (1 μM) + MB-SB2 (5 μM) (Fig. S6).

10.1128/mbio.02239-22.8 FIG S6 Reverse transcription-quantitative PCR (RT-qPCR) analysis of the relative expression of *mbnT* of Methylocystis sp. SB2 growing with 1 μM Cu or with 1 μM Cu + 5 μM MB-SB2. Error bars indicate standard deviations from triplicate biological cultures. *t* tests were performed for variance analysis between the growth conditions. Download FIG S6, DOCX file, 0.02 MB. Copyright © 2022 Peng et al. 2022 Peng et al. https://creativecommons.org/licenses/by/4.0/ This content is distributed under the terms of the Creative Commons Attribution 4.0 International license.

*mmoX* (encoding the 60-kDa α subunit of the sMMO hydroxylase) expression was over 3 orders of magnitude greater in M. trichosporium OB3b wild type, M. trichosporium OB3b Δ*mbnI2R2*, and Δ*mbnT2*-signal domain mutants grown in the absence of copper versus in the presence of copper (1 μM) (*P* < 0.01) (Fig. 3g to i). In the presence of copper + MB-SB2, *mmoX* expression in M. trichosporium OB3b wild type and Δ*mbnI2R2* and Δ*mbnT2*-signal domain mutants increased 1- to 2-fold compared to that found in the presence of 1 μM copper alone. Inversely, *pmoA* (encoding the 27-kDa β subunit of pMMO) expression in M. trichosporium OB3b wild type and Δ*mbnI2R2* and Δ*mbnT2*-signal domain mutants was ~6-fold lower in the absence versus in the presence of copper (*P* < 0.05). In the presence of copper (1 μM) + MB-SB2 (5 μM), *pmoA* expression in these mutants increased 1.5- to 2.5-fold over that observed in the presence of copper alone (*P* < 0.05; Fig. 3k and l). Such upregulation of *pmoA* in the presence of copper + MB-SB2 versus copper alone was not observed in the wild-type M. trichosporium OB3b (Fig. 3j).

These results showed that in the presence of copper and MB-SB2, *mbnT2* expression was induced in wild-type M. trichosporium OB3b. Such *mbnT2* induction was not observed in M. trichosporium OB3b Δ*mbnI2R2* and Δ*mbnT2*-signal domain mutants. However, the two mutants were still able to take up copper in the presence of MB-SB2.

### *mbnT2* expression in M. trichosporium *mbnT1*::Gm^r^.

**FIG 4:**
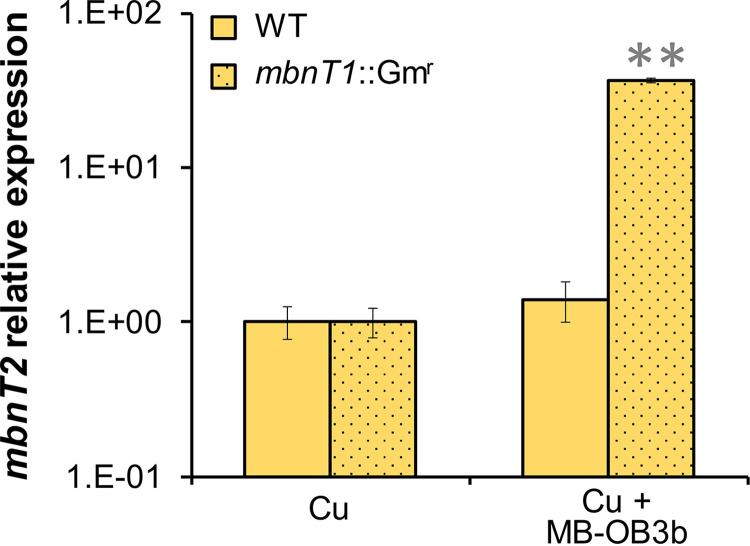
RT-qPCR analysis of the relative expression of *mbnT2* in M. trichosporium OB3b wild type (WT) and *mbnT1*::Gm^r^ mutant growing with 1 μM Cu or 1 μM Cu + 5 μM MB-OB3b. Error bars indicate standard deviations from triplicate biological cultures. *t* tests were performed for variance analysis between the growth conditions. **, *P* < 0.01.

*mbnT2* was also significantly upregulated (36-fold, *P* < 0.01) in a mutant of M. trichosporium OB3b in which the gene encoding the TBDT (MbnT1) responsible for MB-OB3b uptake was knocked out (*mbnT1*::Gm^r^) (33) when grown in the presence of MB-OB3b (5 μM) compared to the absence of MB-OB3b (Fig. 4). In contrast, no significant different in *mbnT2* expression was observed in wild-type M. trichosporium OB3b grown with and without an extra supplement of MB-OB3b (5 μM) (Fig. 4).

### Copper uptake and MMOs expression in M. trichosporium Δ*mbnAN*, Δ*mbnAN* Δ*mbnI2R2*, Δ*mbnAN* Δ*mbnT2*-signal domain, and Δ*mbnAN* Δ*mbnT2* mutants.

To further explore the mechanism of copper uptake in the presence of MB-SB2 by the M. trichosporium Δ*mbnI2R2* and Δ*mbnT2*-signal domain mutants, we created three additional double mutants; i.e., we deleted *mbnI2R2*, the signaling domain region of *mbnT2*, and the entire *mbnT2* gene in a previously constructed mutant of M. trichosporium OB3b defective in production of its own MB-OB3b (i.e., the M. trichosporium Δ*mbnAN* mutant with its MB synthesis genes *mbnABCMN* deleted) (29) (Fig. S4). The deletion of both *mbnAN* and either *mbnI2R2*, *mbnT2*-signal domain, or *mbnT2* was confirmed by PCR (Fig. S5) and sequencing (data not shown).

**FIG 5:**
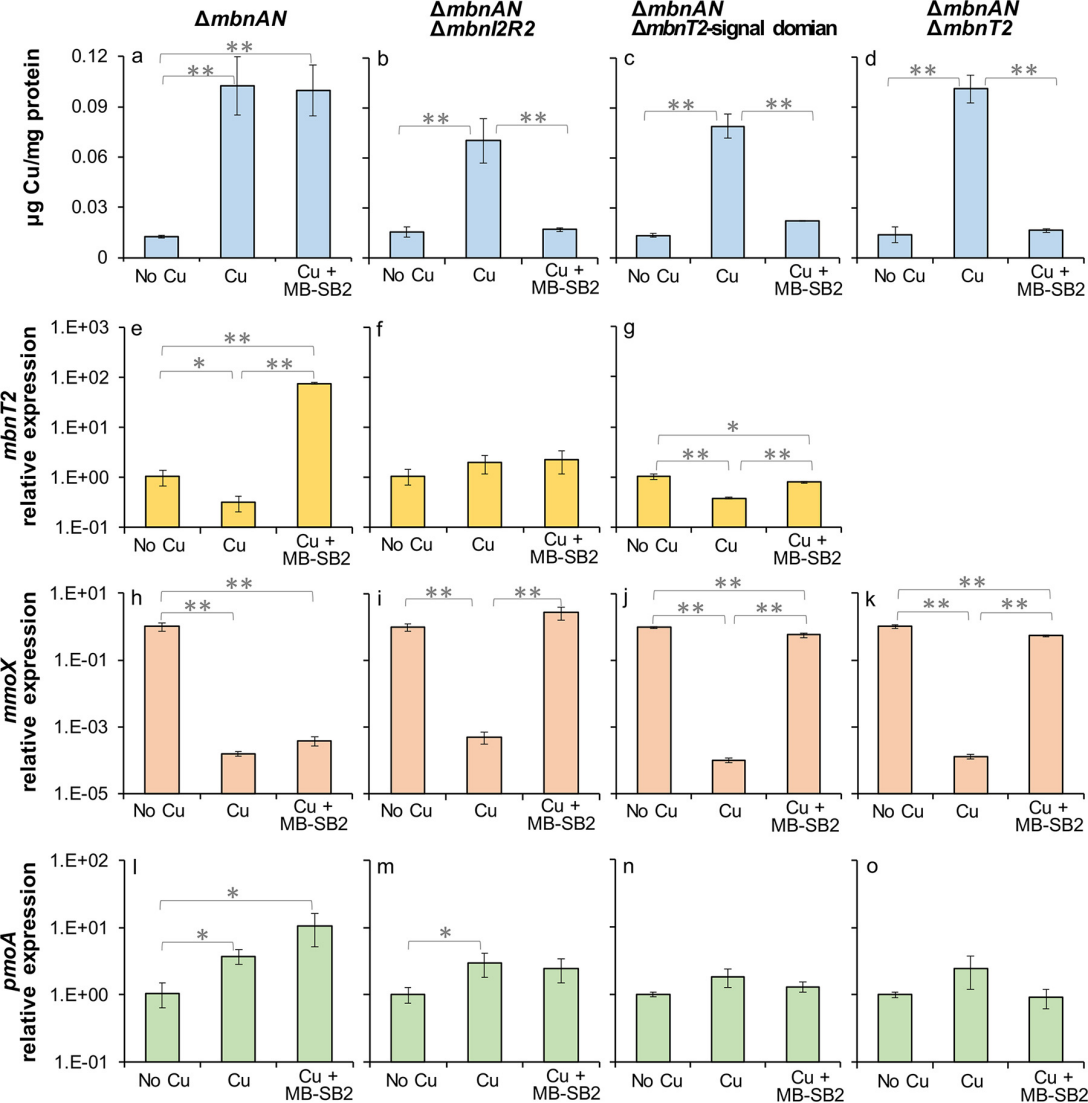
Copper associated with biomass and RT-qPCR analysis of the relative expression of *mbnT2*, *mmoX*, and *pmoA* in M. trichosporium OB3b Δ*mbnAN* (a, e, h, and l), Δ*mbnAN* Δ*mbnI2R2* (b, f, i, and m), Δ*mbnAN* Δ*mbnT2*-signal domain (c, g, j and n), or Δ*mbnAN* Δ*mbnT2* (d, k, and o) mutants growing with no added Cu, with 1 μM Cu, or with 1 μM Cu + 5 μM MB-SB2. Error bars indicate standard deviations from triplicate biological cultures. *t* tests were performed for variance analysis between different growth conditions. *, 0.01 < *P* < 0.05; **, *P* < 0.01.

Copper associated with biomass was 5- to 8-fold higher in M. trichosporium Δ*mbnAN*, Δ*mbnAN* Δ*mbnI2R2*, Δ*mbnAN* Δ*mbnT2*-signal domain, and Δ*mbnAN* Δ*mbnT2* mutants grown with copper versus without copper (*P* < 0.01; Fig. 5a to d). In the presence of copper + MB-SB2, copper associated with biomass in Δ*mbnAN* mutant was comparable to that in the presence of 1 μM copper alone (Fig. 5a), while in the double mutants (Δ*mbnAN* Δ*mbnI2R2*, Δ*mbnAN* Δ*mbnT2*-signal domain, and Δ*mbnAN* Δ*mbnT2*), copper associated with biomass decreased to that observed in the absence of copper (Fig. 5a to d).

Similar to wild-type M. trichosporium OB3b, *mbnT2* was significantly upregulated (75- to 240-fold; *P* < 0.01) in M. trichosporium Δ*mbnAN* mutant when grown in the presence of copper + MB-SB2 compared to the absence or presence of 1 μM copper alone (Fig. 5e). Such upregulation was not observed in M. trichosporium OB3b Δ*mbnAN* Δ*mbnI2R2* mutant, in which *mbnT2* expression was invariant when grown with or without copper and/or MB-SB2 (Fig. 5f). *mbnT2* expression was uniformly low in the Δ*mbnAN* Δ*mbnT2*-signal domain mutant under all conditions but was significantly (2- to 3-fold) less when this mutant was grown in the presence of copper versus either the absence of copper or copper added in the presence of + MB-SB2 (5 μM) (Fig. 5g).

*mmoX* expression was 3 to 4 orders of magnitude higher in M. trichosporium Δ*mbnAN*, Δ*mbnAN* Δ*mbnI2R2*, Δ*mbnAN* Δ*mbnT2*-signal domain, and Δ*mbnAN* Δ*mbnT2* mutants grown in the absence of copper versus presence of 1 μM copper (*P* < 0.01). In the presence of copper + MB-SB2, *mmoX* expression in Δ*mbnAN* decreased to the same level of that in the presence of copper, while for the other double mutants, the *mmoX* expression level was equivalent to that observed in the absence of copper (Fig. 5h to k).

*pmoA* expression in the M. trichosporium Δ*mbnAN*, Δ*mbnAN* Δ*mbnI2R2*, Δ*mbnAN* Δ*mbnT2*-signal domain, and Δ*mbnAN* Δ*mbnT2* mutants decreased 2- to 3-fold in the absence versus in the presence of copper. In the presence of copper + MB-SB2, *pmoA* expression in Δ*mbnAN* was comparable to that with copper (1 μM) alone. *pmoA* expression slightly decreased (1.2- to 2.5-fold) in all double mutants when grown with copper + MB-SB2 versus copper alone (Fig. 5l to o), although such differences were not significant (*P* > 0.05). Collectively, these results indicated that copper bound to MB-SB2 was unavailable to M. trichosporium mutants defective in both MB-OB3b production and MbnT2 induction/function. Moreover, native MB production was essential for copper uptake in M. trichosporium mutants in which either *mbnT2/mbnI2R2* has been deleted or the internal signaling domain has been removed.

### Copper transfer/extraction between MB-OB3b and MB-SB2.

**FIG 6:**
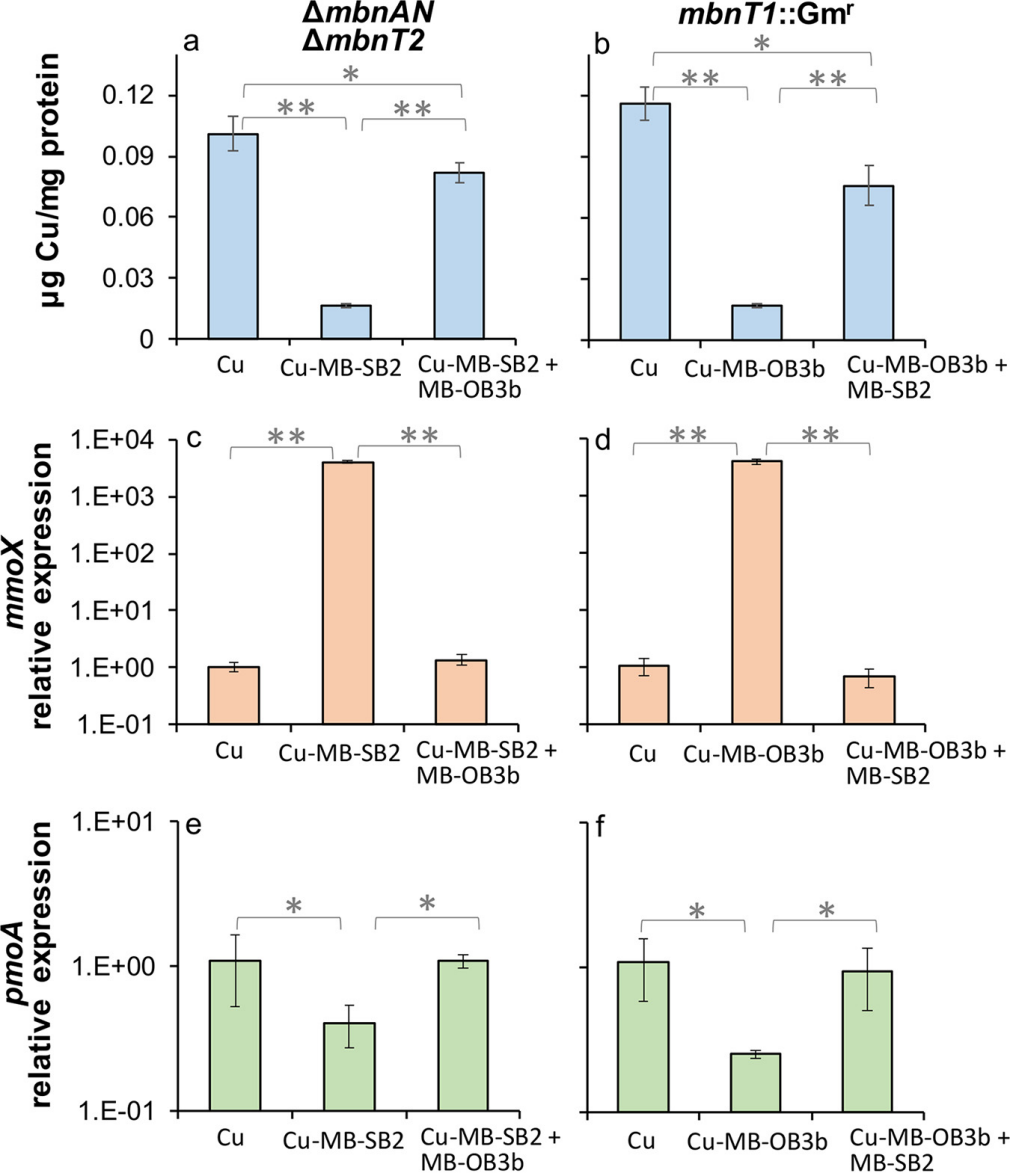
Copper associated with biomass and RT-qPCR analysis of the relative expression of *mmoX* and *pmoA* in M. trichosporium Δ*mbnAN* Δ*mbnT2* (a, c, and e) and *mbnT1*::Gm^r^ (b, d, and f) mutants growing with 1 μM Cu, 1 μM Cu-MB-SB2/OB3b or 1 μM Cu-MB-SB2/OB3b + 5 μM MB-OB3b/SB2. Error bars indicate standard deviations from triplicate biological cultures. *t* tests were performed for variance analysis between different growth conditions. *, 0.01 < *P* < 0.05; **, *P* < 0.01.

To investigate how MB-OB3b can facilitate copper uptake by the M. trichosporium mutants that cannot properly regulate *mbnT2* expression (i.e., Δ*mbnI2R2* and Δ*mbnT2*-signal domain mutants), we characterized the ability of the M. trichosporium Δ*mbnAN* Δ*mbnT2* to collect copper in the presence of exogenous MB-OB3b. When Δ*mbnAN* Δ*mbnT2* was grown in the presence of copper-loaded MB-SB2 (Cu-MB-SB2) and MB-OB3b (see Materials and Methods for a detailed description of experimental protocols), copper associated with biomass was ~5-fold higher (*P* < 0.01) than in the absence of added MB-OB3b (Fig. 6a). In addition, *mmoX* expression was over 3 orders of magnitude lower (*P* < 0.01) in the presence of MB-OB3b, while *pmoA* expression was 2.5-fold higher (*P* < 0.05) (Fig. 6c and e). Moreover, copper associated with biomass in Δ*mbnAN* Δ*mbnT2* was only slightly (~20%) (*P* < 0.05) lower when grown with Cu-MB-SB2 + MB-OB3b (5 μM) versus copper alone (Fig. 6a). *mmoX* and *pmoA* expression were comparable when growing this mutant with Cu-MB-SB2 + MB-OB3b versus copper alone (Fig. 6c and e). These results suggest that MB-OB3b can extract copper from Cu-MB-SB2. We further investigated this to determine whether MB-SB2 can extract copper from Cu-MB-OB3b in M. trichosporium OB3b *mbnT1*::Gm^r^ that cannot take up copper when bound to MB-OB3b (Cu-MB-OB3b) (33). When *mbnT1*::Gm^r^ was growing in the presence of Cu-MB-OB3b + MB-SB2, copper associated with biomass was 4.7-fold higher (*P* < 0.01), *mmoX* expression was over 3 orders of magnitude lower (*P* < 0.01), and *pmoA* expression was 3.8-fold higher (*P* < 0.05) versus growing with Cu-MB-OB3b alone (Fig. 6b, d, and f). Moreover, copper associated with biomass in *mbnT1*::Gm^r^ was ~30% less (*P* < 0.05) when grown with Cu-MB-OB3b + MB-SB2 versus copper alone (Fig. 6a). *mmoX* and *pmoA* expression was comparable when growing this mutant with Cu-MB-OB3b + MB-SB2 versus copper alone (Fig. 6d and f). These results further indicate that MB-SB2 can extract copper from Cu-MB-OB3b; i.e., it appears that MB-OB3b and MB-SB2 can extract copper from the Cu-MB complex of their counterpart.

Previous studies have shown the copper affinity for both MB-OB3b and MB-SB2 varies depending on copper to MB molar ratio (16, 42). To illustrate copper binding by either form of MB, UV-visible spectrophotometry provides a simple and convenient assay as the magnitude and wavelength of absorption maxima of the heterocyclic rings decreases with increasing copper (Fig. S7A and B). That is, a 10-nm blue shift is observed in the absorption maxima of the C-terminal oxazolone ring of both MB-OB3b and MB-SB2 (344 to 334 nm and 336 to 326 nm, respectively) (Fig. S7A and B) as copper is added. These assays can be extended to monitor copper exchange between MB-OB3b and MB-SB2. The addition of metal free MB-OB3b to an 80% saturated solution of MB-SB2 resulted in an incremental red shift in the oxazolone group of MB-SB2, demonstrating copper loss by MB-SB2 and copper uptake by MB-OB3b (Fig. S7C). Conversely, the incremental addition of metal free MB-SB2 to an 80% copper saturated solution of MB-OB3b resulted in an incremental blue shift in the oxazolone group of MB-SB2, demonstrating copper uptake by MB-SB2 from copper containing MB-OB3b (Fig. S7D).

10.1128/mbio.02239-22.9 FIG S7 (A) UV-visible absorption spectra of 50 nmol mL^−1^ (50 μM) MB-OB3b solution (black line) following the addition of 5 nmol (orange line), 10 nmol (gray line), 15 nmol (yellow line), 20 nmol (light blue line), 25 nmol (green line), 30 nmol (dark blue line), 35 nmol (gold line), or 40 nmol (red line) CuCl_2_. (B) UV-visible absorption spectra of 50 nmol mL^−1^ (50 μM) MB-SB2 solution (black line) following the addition of 5 nmol (gray line), 10 nmol (orange line), 15 nmol (light blue line), 20 nmol (blue line), 25 nmol (green line), 30 nmol (gold line), 35 nmol (dark blue line), or 40 nmol (red line) CuCl_2_. (C) UV-visible absorption spectra of 50 nmol mL^−1^ (50 μM) MB-SB2 plus 40 nmol CuCl_2_ (red line) following the addition of 15 nmol MB-OB3b (green line), 30 nmol MB-OB3b (blue line), or 45 nmol MB-OB3b (black line). (D) UV-visible absorption spectra of 50 nmol mL^−1^ (50 μM) MB-OB3b plus 40 nmol CuCl_2_ (red line) following the addition of 15 nmol MB-SB2 (green line), 30 nmol MB-SB2 (blue line), or 45 nmol MB-SB2 (black line). Download FIG S7, PDF file, 0.4 MB. Copyright © 2022 Peng et al. 2022 Peng et al. https://creativecommons.org/licenses/by/4.0/ This content is distributed under the terms of the Creative Commons Attribution 4.0 International license.

## DISCUSSION

In an earlier study (34), we identified a second MB uptake system (mediated by MbnT2) in M. trichosporium OB3b that is responsible for uptake of heterologous MB produced from Methylocystis sp. SB2 and determined that the expression of *mbnT2* is induced by MB-SB2. Here, we show that such upregulation is dependent on a signaling domain at the N terminus of MbnT2. It appears that this domain acts in concert with an ECF sigma factor and an anti-sigma regulator (MbnI2 and MbnR2, respectively) that are encoded by genes immediately upstream of *mbnT2*. Such a signaling domain is absent in MbnT of Methylocystis sp. SB2, as well as these sigma and anti-sigma factor-encoding genes not associated with *mbnT* in Methylocystis sp. SB2 (28). This suggests that MbnT expression in Methylocystis sp. SB2 is not regulated by MB-SB2, as was found in M. trichosporium OB3b. Indeed, MbnT expression in Methylocystis sp. SB2 is not upregulated by the exogenous addition of MB-SB2 (Fig. S6).

**FIG 7:**
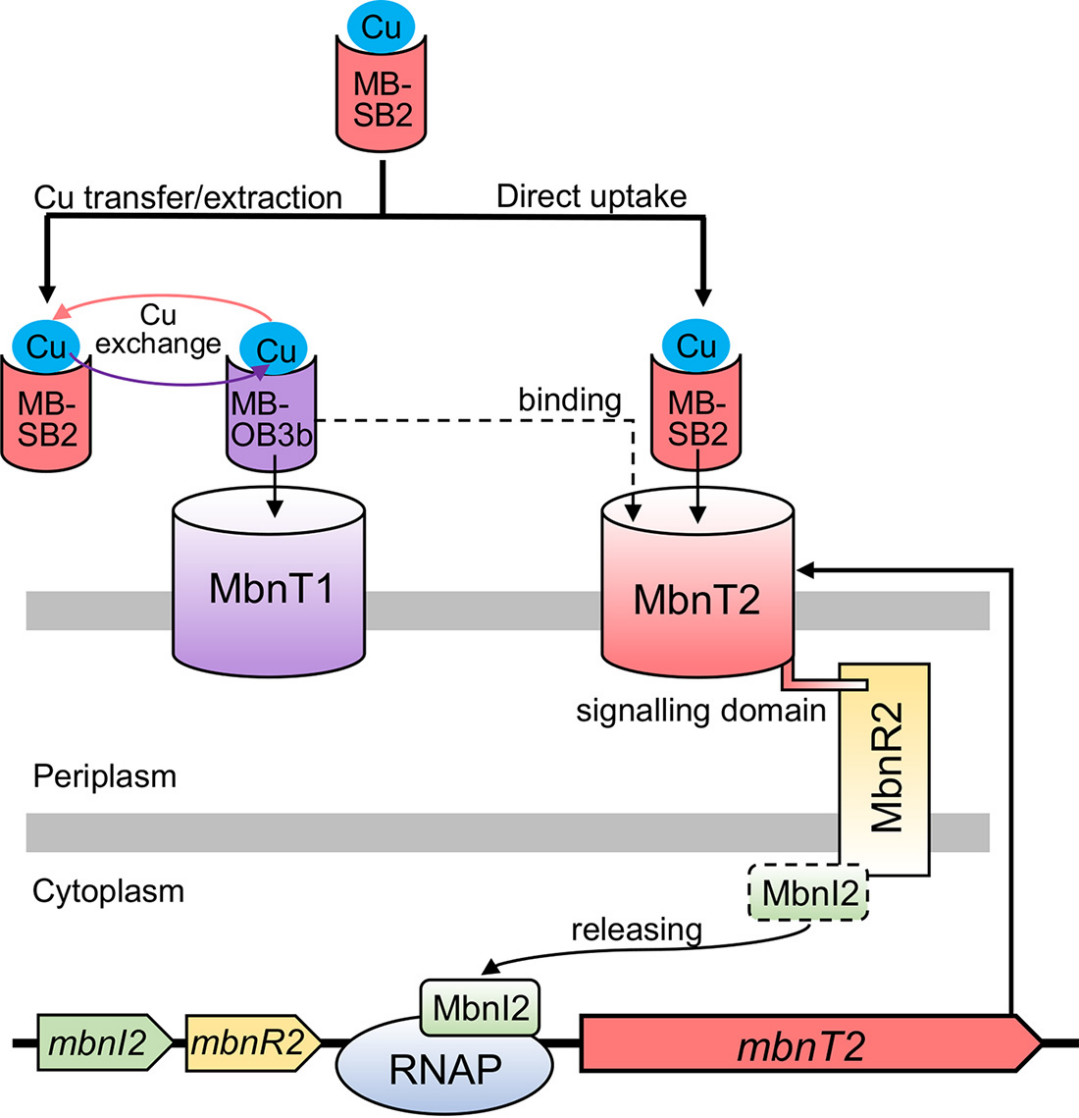
Proposed mechanisms for copper uptake from Cu-MB-SB2 by M. trichosporium OB3b via either copper exchange between MB-OB3b and MB-SB2 or initiating transcription of MbnT2 that is mediated by the signaling domain of MbnT2 and ECF sigma factor (MbnI2) and anti-sigma factor (MbnR2). MbnI2 is released from MbnR2 to recruit RNA polymerase (RNAP) upon binding of MB-SB2 or MB-OB3b to MbnT2 that generates a signal transmitted to MbnR2.

TBDT and ECF sigma factor and anti-sigma regulator-mediated gene regulation has been extensively studied in microbial ferric-siderophore uptake systems (36, 37). Accordingly, the induction of gene expression by MB-SB2 in M. trichosporium OB3b can be proposed as follows (Fig. 7): (i) the binding of MB-SB2 to MbnT2 causes a conformational change of the barrel and plug domains of MbnT2; (ii) the signaling domain of MbnT2 then transduces the signal by interacting with the C-terminal domain of the inner membrane anti-sigma factor MbnR2; (iii) MbnR2 subsequently releases the ECF sigma factor MbnI2 into the cytoplasm to recruit the core RNA polymerase (RNAP); and finally, (iv) MbnI2 promotes binding of the RNAP to the promoter of *mbnT2*, thereby initiating transcription.

It has been demonstrated that ferric-siderophore binding to the corresponding TBDT alone is sufficient for signal generation and transcriptional induction. That is, transport of the ferric-siderophore is not necessary for the signal transmission process (39, 43, 44). Interestingly, we found *mbnT2* can also be induced in M. trichosporium *mbnT1*::Gm^r^ in the presence of MB-OB3b (Fig. 4) (*mbnT1*::Gm^r^ is unable to take up MB-OB3b due to the disruption of MbnT1). Such MB-OB3b-initialized *mbnT2* induction is likely due to the nonspecific binding of MB-OB3b to MbnT2. It has been previously found that MB-OB3b can not only bind to MbnT1 that is responsible for MB-OB3b uptake (32, 33) but also can bind to MbnT of Methylocystis rosea SV97 (MbnT-SV97) (32). MbnT2 shares high identity (57%) and similarity (E value: 0) with MbnT-SV97 (moreover, the overall identity of MbnT-SV97 and MbnT-SB2 is 93%) (data not shown). Due to the high similarity between MbnT2, MbnT-SV97, and MbnT-SB2, it is likely that MB-OB3b can also bind to MbnT2 and thereby generate a signal to initialize the transcription of *mbnT2*. MB-OB3b initialized transcription of *mbnT2* is not observed in wild-type M. trichosporium OB3b, indicating specific binding (and uptake) of MB-OB3b to its cognate transporter MbnT1 rather than weaker nonspecific binding of MB-OB3b to MbnT2 in wild-type M. trichosporium OB3b. It should be noted that MbnT2 cannot take up/transport MB-OB3b as MbnT1 is the only TBDT for MB-OB3b uptake in M. trichosporium OB3b (33) (Fig. 6b).

An N-terminal signaling domain is also found in MbnT1 of M. trichosporium OB3b. Moreover, ECF sigma and anti-sigma factors encoding genes (*mbnI1* and *mbnR1*) are also adjacent to *mbnT1* (Fig. 1). Due to the similar structure of MbnT1 and MbnT2, as well as the genetic organization of the *mbnT1* and *mbnT2* gene clusters, it is reasonable to speculate that binding of MB-OB3b to MbnT1 should also generate a signal to release MbnI1 and thereby initialize transcription of MbnT1. However, unlike MbnT2, MbnT1 expression is not controlled by MB-OB3b (34). Rather, MbnT1 is constitutively expressed. That is, the expression of MbnT1 is invariant in M. trichosporium OB3b grown with and without exogenous MB-OB3b (34). MbnI1 might be responsible for regulating expression of other functional genes rather than *mbnT1*. Previous studies indicate that the regulon of TBDT-mediated ECF sigma factor is rather broad (40). For example, the siderophore uptake system of Pseudomonas aeruginosa PAO1 consists of a siderophore TBDT receptor (FpvA), an anti-sigma factor FpvR, and the two ECF sigma factors FpvI and PvdS. In this cascade, the binding of siderophore transmits a signal to release FpvI and PvdS, which control the expression of over 80 genes, including *fpvA* (the ferric-siderophore transporter encoding gene), ferric-siderophore biosynthesis genes, transporter genes for heme uptake, and small RNA genes (40, 42, 45). Hence, it is possible that MbnI1 is involved in regulating as-of-yet-unknown gene(s) in M. trichosporium OB3b. Moreover, in addition to regulating *mbnT2* expression, MbnI2 might also be involved in regulating other (yet-unknown) functional gene(s). Further research is clearly needed to address these questions.

Herein, we show that MbnI2, MbnR2, and MbnT2 signaling domain-mediated transcriptional induction of MbnT2 is essential for MB-SB2 uptake by M. trichosporium OB3b. As demonstrated previously (34) and together with this study, MbnT2 is the only TBDT responsible for MB-SB2 transport/uptake in M. trichosporium OB3b. However, copper bound to MB-SB2 (Cu-MB-SB2) is still available for M. trichosporium OB3b mutants that cannot properly regulate *mbnT2* expression (Fig. 3). The production of MB-OB3b apparently plays a key role in copper uptake from Cu-MB-SB2 by the mutants defective in *mbnT2* expression or function, as deletion of *mbnAN* genes in these mutants disrupted their ability to take up copper from Cu-MB-SB2 (Fig. 5). That is, in the M. trichosporium OB3b mutant that can neither take up MB-SB2 nor produce MB-OB3b (i.e., the Δ*mbnAN* Δ*mbnT2* mutant), adding MB-OB3b enabled this mutant to extract copper from Cu-MB-SB2 (Fig. 6a, c, and e). Likewise, in a M. trichosporium OB3b mutant that cannot take up MB-OB3b (i.e., *mbnT1*::Gm^r^ mutant), adding MB-SB2 enables this mutant to extract copper from Cu-MB-OB3b (Fig. 6b, d and f). Intriguingly, the exchange of copper between different forms of MB can occur, but such an exchange is concentration dependent (Fig. S7C and D). Clearly, interactions between methanotrophs for copper and competition for copper binding by MBs are much more complicated than initially presumed, and much more work is warranted to investigate these phenomena.

In any regard, copper uptake using MBs is an important mechanism for methanotrophs to collect this essential trace element from the environment lack of bioavailable copper. For example, MB-OB3b can extract copper from copper-sulfide minerals typically assumed to be biologically unavailable (46). Here, we demonstrate that the model methanotroph M. trichosporium OB3b can not only secrete and uptake its own MB but also has developed multiple mechanisms to take up copper in the presence of heterologous methanobactin MB-SB2, including initiating transcription of an alternative TBDT. A survey of available methanotrophic genomes shows a similar cooccurrence of alternative TBDTs and regulatory systems for heterologous MB uptake among MB-producing methanotrophs, especially group I MB-producing methanotrophs (Table S1). Interestingly, signaling domains are also found in TBDTs potentially responsible for heterologous MBs uptake in methanotrophs that produce group I MB, and sigma and anti-sigma encoding genes are adjacent to the TBDT genes (Table S1). Such genetic features suggest a similar regulatory mechanism of these TBDTs compared to MbnT2 in M. trichosporium OB3b. Moreover, all currently identified group I MB-producing methanotrophs express alternative MMOs (i.e., pMMO and sMMO). Together with the MbnT (responsible for their own/homologous MB uptake) of these methanotrophs, these TBDTs may also be involved in regulating the expression of alternative MMOs (i.e., the canonical “copper switch”) in these methanotrophs as previously demonstrated in M. trichosporium OB3b (i.e., MbnT1 and MbnT2 comedicated copper switch) (34). Further studies are needed to investigate the regulation and functions of these TBDTs in MB uptake and methane metabolism (i.e., copper switch).

10.1128/mbio.02239-22.1 TABLE S1 Potential TonB-dependent transporters (TBDTs) in methanobactin (MB)-producing methanotrophs for heterologous and/or homologous MB uptake. Download Table S1, DOCX file, 0.03 MB. Copyright © 2022 Peng et al. 2022 Peng et al. https://creativecommons.org/licenses/by/4.0/ This content is distributed under the terms of the Creative Commons Attribution 4.0 International license.

## MATERIALS AND METHODS

### MB isolation.

MBs from M. trichosporium OB3b and Methylocystis sp. strain SB2 were isolated from their spent media as previously described by Bandow et al. (47). The purity of the isolated methanobactins was determined by high-performance liquid chromatography (HPLC) as described previously (48).

### Growth conditions.

M. trichosporium OB3b and constructed mutants (Table 1) were grown in nitrate mineral salt (NMS) medium (26) with or without 1 μM CuCl_2_. Methylocystis sp. SB2 was grown in NMS medium with 1 μM CuCl_2_. Methane and air were added at a methane-to-air ratio of 1:2. The cultures were incubated in dark at 30°C. Liquid cultures were grown in 250-mL sidearm Erlenmeyer flasks with 20 to 30 mL NMS medium with shaking at 200 rpm. MB from M. trichosporium OB3b or Methylocystis sp. SB2 were filter sterilized and added to NMS medium at final concentration of 5 μM as described previously (49). Solid NMS medium was supplemented with 1.2% agar. Growth was monitored by measuring the optical density at 600 nm (OD_600_) with a Genesys 20 visible spectrophotometer (Spectronic Unicam, Waltham, MA). Triplicate biological cultures were harvested at middle to late exponential phase for OD_600_ measurement, transcriptional analysis of specific gene expression, and metal distribution. Escherichia coli was grown in Luria-Bertani broth (LB) at 37°C with or without a supplement of 25 μg/mL kanamycin.

**TABLE 1:** Bacterial strains used in this study

Strain	Description/genotype	Reference
M. trichosporium OB3b	Wild type	26
Methylocystis sp. strain SB2	Wild type	60
M. trichosporium *mbnT1*::Gm^r^	*mbnT1* marker exchanged mutant with gentamicin resistance gene	33
M. trichosporium *ΔmbnAN*	*mbnAN* deleted	29
M. trichosporium *ΔmbnT2*	*mbnT2* deleted	34
M. trichosporium *ΔmbnI2R2*	*mbnI2R2* deleted	This work
M. trichosporium *ΔmbnT2*-signal domain	*mbnT2* signal domain encoding region deleted	This work
M. trichosporium *ΔmbnAN ΔmbnI2R2*	*mbnAN* and *mbnI2R2* deleted	This work
M. trichosporium *ΔmbnAN ΔmbnT2*-signal domain	*mbnAN* and *mbnT2* signal domain encoding region deleted	This work
M. trichosporium *ΔmbnAN ΔmbnT2*	*mbnAN* and *mbnT2* deleted	This work
E. coli TOP10	Strain used for plasmid construction and cloning. F^−^ *mcrA* Δ(*mrr-hsdRMS-mcrBC*) Φ80*lacZ*ΔM15 Δ*lacX*74 *recA1 araD139* Δ(*ara leu*) 7697 *galU galK rpsL* (Str^r^)	Invitrogen
E. coli S17-1	Conjugative donor; *recA1 thi pro hsdR*-RP4-2Tc::Mu Km::Tn7	51

### Construction of the M. trichosporium OB3b Δ*mbnI2R2*, Δ*mbnT2*-signal domain mutants.

*mbnI2R2* and *mbnT2*-signal domain were deleted in M. trichosporium OB3b wild type using a previously described protocol (50) with modifications. Briefly, upstream and downstream regions of the respective gene (arms A and B, respectively) were PCR amplified using the arm primers listed in Table S2. Arms A and B were digested with the restriction enzymes and ligated together to form arm AB, which was subsequently inserted into the pK18mob*sacB* mobilizable suicide vector (Fig. S4) (51). The pK18mob*sacB* vector with arm AB was transferred to E. coli TOP10 (Invitrogen, Carlsbad, CA). Plasmid was extracted from transformed E. coli Top10 using a plasmid mini kit (Qiagen, Hilden, Germany) following the manufacturer’s instructions. The extracted plasmid was then transferred to E. coli S17-1 (52). Conjugation of E. coli S17-1 carrying the constructed vector with M. trichosporium OB3b was performed as described by Martin and Murrell (53). Transconjugants were grown on NMS plates supplemented with 25 μg/mL kanamycin and 10 μg/mL nalidixic acid. One kanamycin-resistant transconjugant colony (generated after 10 days incubation) was transferred to an NMS plate with kanamycin (25 μg/mL), incubated for 7 days, and subsequently transferred to an NMS plate with 2.5% sucrose (mass/volume). Sucrose-resistant colonies were generated after 10 days of incubation and were screened for mutation with deletion of *mbnI2R2* and *mbnT2*-signal domain by colony PCR using the checking primers (Table S2). Successful deletion mutation was further confirmed by PCR with DNA extracted from the mutant using the DNeasy PowerSoil Pro kit (Qiagen, Hilden, Germany) following the manufacturer’s instructions.

10.1128/mbio.02239-22.2 TABLE S2 Primers used in this study. Download Table S2, DOCX file, 0.02 MB. Copyright © 2022 Peng et al. 2022 Peng et al. https://creativecommons.org/licenses/by/4.0/ This content is distributed under the terms of the Creative Commons Attribution 4.0 International license.

### Construction of the M. trichosporium OB3b Δ*mbnAN* Δ*mbnI2R2*, Δ*mbnAN* Δ*mbnT2*-signal domain, and Δ*mbnAN* Δ*mbnT2* mutants.

To construct M. trichosporium OB3b mutants with double deletion of MB synthesis genes and *mbnI2R2* or *mbnT2*-signal domain or *mbnT2*, the previously constructed M. trichosporium OB3b mutant with its MB biosynthesis genes *mbnABCMN* deleted (Δ*mbnAN* mutant) (29) was used as the conjugation acceptor. That is, *mbnI2R2*, *mbnT2*-signal domain, and *mbnT2* were deleted in M. trichosporium OB3b Δ*mbnAN* mutant. The pK18mob*sacB* vector with arms targeting the homologous regions of *mbnI2R2*, *mbnT2*-signal domain, and *mbnT2* were obtained from this study (described above) or from the previous study (34). Conjugation, growing of the transconjugates, and selection of the double deletion mutants were performed as outlined above.

### Copper competition and transfer between MBs.

Copper-bound MB complexes (Cu-MB-OB3b/SB2) were prepared by mixing 1 μM CuCl_2_ and 5 μM MB-OB3b/SB2 in NMS medium followed by incubating in the dark at 30°C with shaking at 200 rpm for 1 h. M. trichosporium Δ*mbnAN* Δ*mbnT2* (this study) and *mbnT1*::Gm^r^ mutants (33) were used for the study of copper competition and transfer between MBs. Δ*mbnAN* Δ*mbnT2* and *mbnT1*::Gm^r^ were grown in NMS medium containing 1 μM Cu-MB-SB2 + 5 μM MB-OB3b (Δ*mbnAN* Δ*mbnT2* mutant) or Cu-MB-OB3b + 5 μM MB-SB2 (*mbnT1*::Gm^r^ mutant). Δ*mbnAN* Δ*mbnT2* and *mbnT1*::Gm^r^ were also grown in NMS medium containing 1 μM Cu alone as positive controls and grown in 1 μM Cu-MB-SB2 alone (Δ*mbnAN* Δ*mbnT2* mutant) or 1 μM Cu-MB-OB3b alone (*mbnT1*::Gm^r^ mutant) as negative controls. Cells of the mutants were collected at the middle to late exponential growth phase for RNA isolation and copper measurement.

Copper titration of MB-OB3b and MB-SB2 were determined as described by Choi et al. (16) and Bandow et al. (54), respectively. To obtain 80% copper saturated 50 nmol (50 μM) MB-OB3b or 50 nmol (50 μM) MB-SB2 were mixed with 40 nmol (40 μM) CuCl_2_ and determined by the UV-visible absorption spectra. Copper extraction from 80% copper saturated MB-OB3b or MB-SB2 was determined by monitoring the UV-visible spectral changes in the oxazolone group of MB-SB2 following the addition of MB-SB2 or MB-OB3b.

### RNA isolation and reverse transcription-quantitative PCR (RT-qPCR).

RNA isolation was performed with a bead-beating procedure followed by column purification using an RNeasy mini kit (Qiagen, Hilden, Germany) as described before (55). Genomic DNA was removed from the column with RNase-free DNase (Qiagen, Hilden, Germany) treatment. The absence of genomic DNA was confirmed by 16S rRNA gene targeted PCR with extracted RNA samples as the templates. The purified RNA was quantified using a NanoDrop 1000 spectrophotometer (Thermo Scientific, Wilmington, DE). cDNA was synthesized from 200 ng total RNA using SuperScript III reverse transcriptase (Invitrogen, Carlsbad, CA) following the manufacturer’s instructions.

RT-qPCR assays were performed to determine the relative expression of the *mbnT2*, *pmoA*, and *mmoX* in M. trichosporium OB3b and mutant strains grown in the presence or absence of copper and/or methanobactins. RT-qPCR was performed using the iTaq Universal SYBR green Supermix (Bio-Rad, Hercules, CA) with 96-well PCR plates on a CFX Connect real-time PCR detection system (Bio-Rad, Hercules, CA). The RT-qPCR program was: 95°C for 10 min, followed by 40 cycles of 95°C for 15 s, 56°C for 30 s, and 72°C for 30 s. Melting curves were measured from 65 to 95°C with increments of 0.5°C and 10 s at each step. Transcription of the targeted genes was determined using cDNA as the template. The transcript levels were calculated by relative quantification using the 2^−ΔΔCT^ method (56) with the 16S rRNA gene as the reference gene (57).

### Metal analysis.

Cells of M. trichosporium OB3b and the mutant strains grown under different conditions were collected and acid digested as described previously (33, 57). Copper associated with biomass was subsequently analyzed using an inductively coupled plasma mass spectrometer (ICP-MS; Agilent Technologies, Santa Clara, CA).

### Data availability.

The materials and data generated in this study will be made available upon reasonable request to the corresponding author.
