# Supplementary material for: Multiple Mechanisms for Copper Uptake by Methylosinus trichosporium OB3b in the Presence of Heterologous Methanobactin
Source: mBio. 2022 Sep 21;13(5):e02239-22. doi: 10.1128/mbio.02239-22 (PMC9601215; doi:10.1128/mbio.02239-22)
Supplement: FIG S1 [file mbio.02239-22-s0003.docx]

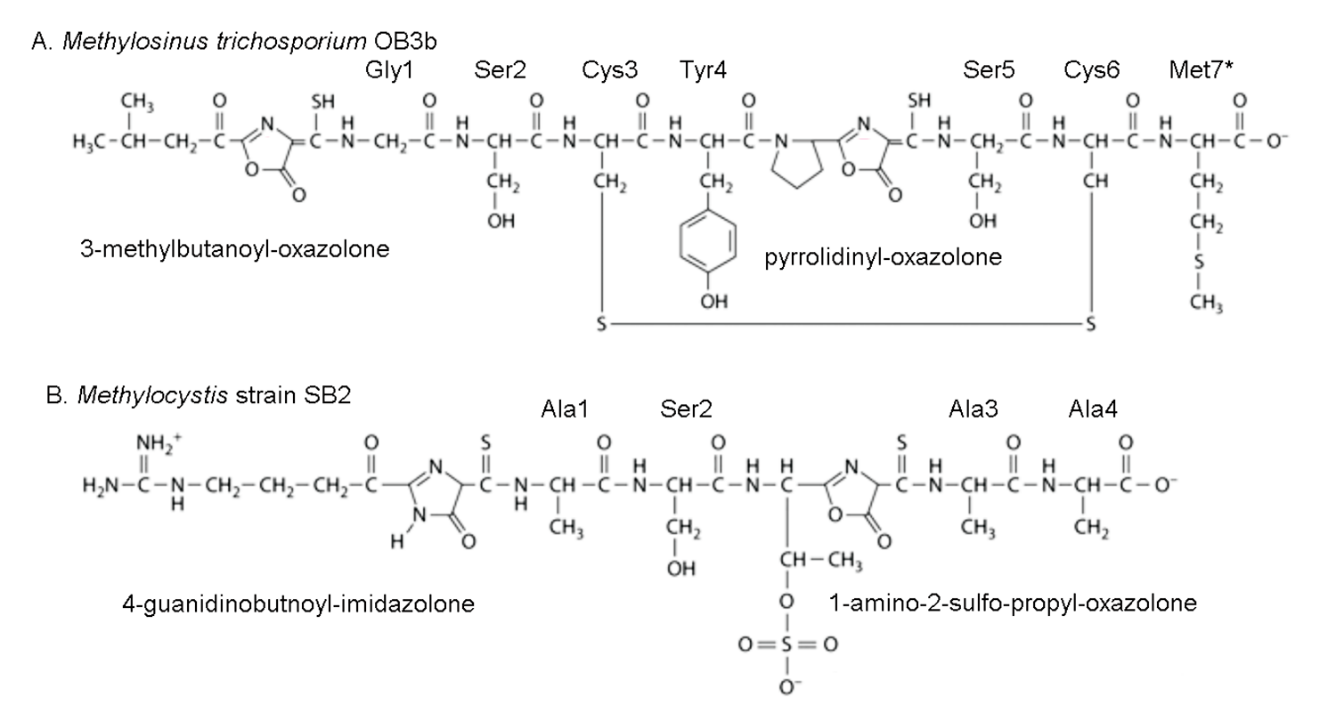


**Fig S1**. Primary structures of methanobactin from (A) *M. trichosporium* OB3b and (B) *Methylocystis* sp. strain SB2.
