# Supplementary material for: Multiple Mechanisms for Copper Uptake by Methylosinus trichosporium OB3b in the Presence of Heterologous Methanobactin
Source: mBio. 2022 Sep 21;13(5):e02239-22. doi: 10.1128/mbio.02239-22 (PMC9601215; doi:10.1128/mbio.02239-22)
Supplement: FIG S2 [file mbio.02239-22-s0004.docx]

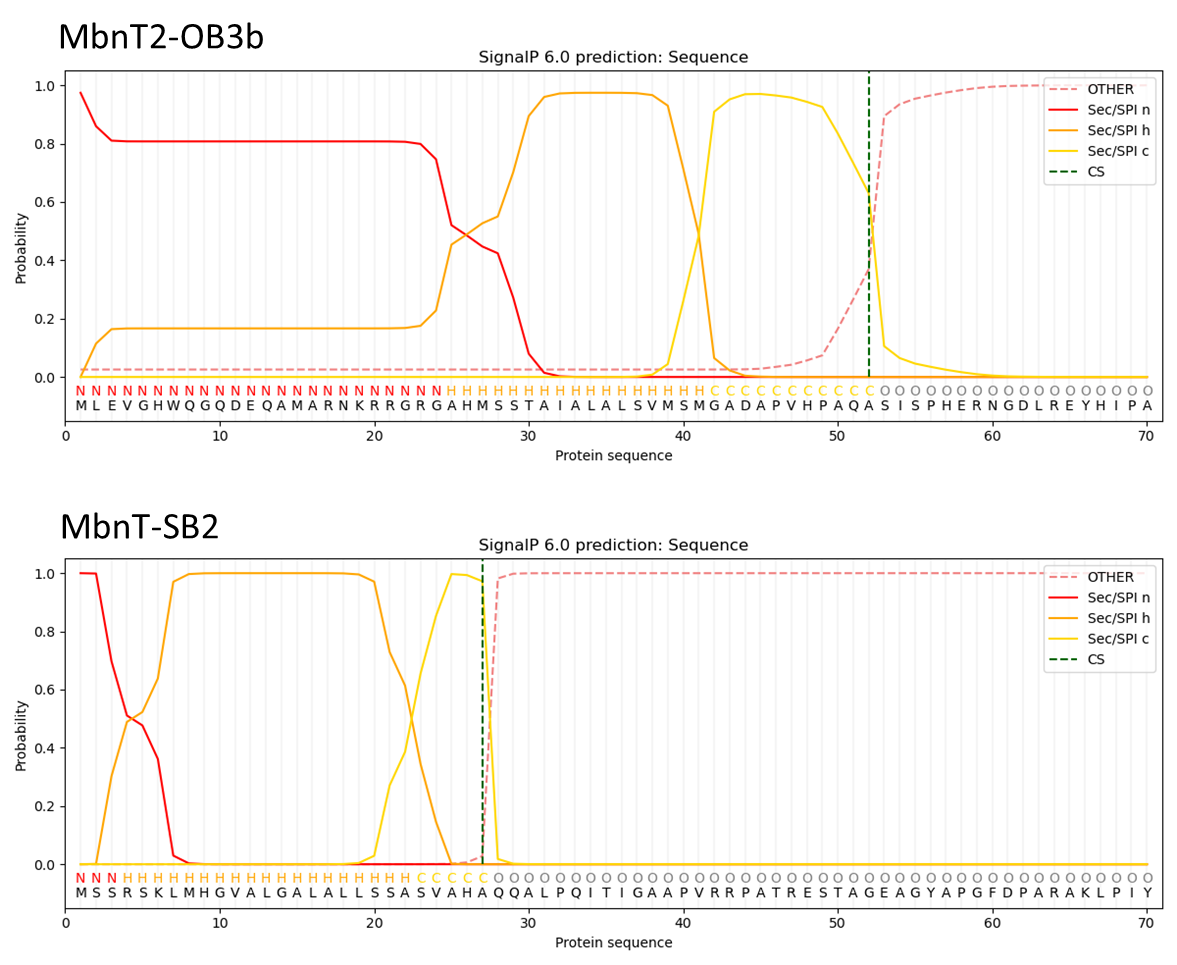


**Fig S2.** Signal peptide (SP) and cleavage site (CS) prediction for the MbnT2 of *M. trichosporium* OB3b and MbnT of *Methylocystis* sp. SB2 using SignalP-6.0 that can predict all known types of SPs based on protein language models (Teufel et al,. 2022). The N-terminal region, hydrophobic region, and C-terminal region of the signal peptide was labeled as “N” (marked in red), “H” (marked in orange), and “C” (marked in yellow), respectively. The CS site is indicated with a green dashed line. Reference: Teufel F, Almagro Armenteros JJ, Johansen AR, Gíslason MH, Pihl SI, Tsirigos KD, Winther O, Brunak S, von Heijne G, Nielsen H. 2022. SignalP 6.0 predicts all five types of signal peptides using protein language models. Nat Biotechnol:1-3.
