# Supplementary material for: Multiple Mechanisms for Copper Uptake by Methylosinus trichosporium OB3b in the Presence of Heterologous Methanobactin
Source: mBio. 2022 Sep 21;13(5):e02239-22. doi: 10.1128/mbio.02239-22 (PMC9601215; doi:10.1128/mbio.02239-22)
Supplement: FIG S3 [file mbio.02239-22-s0005.docx]

**
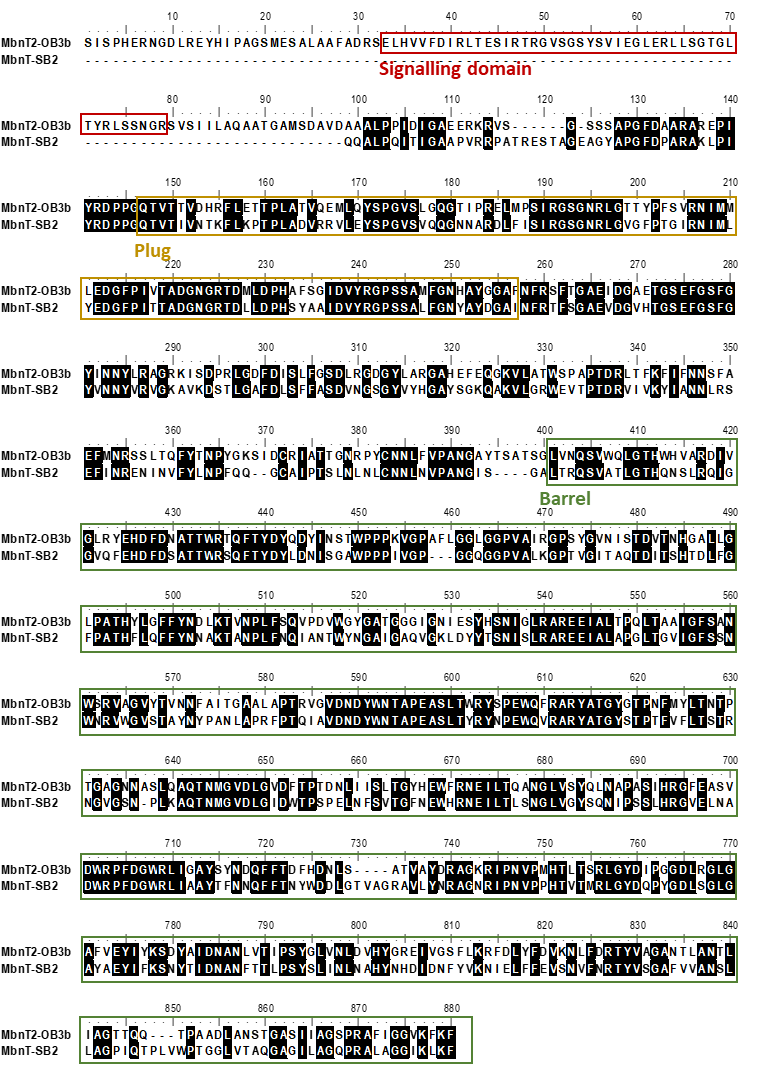
**

**Fig S3.** T-Coffee multiple sequence alignment (Notredame et al., 2000) of mature MbnT2 of *M. trichosporium* OB3b and MbnT of *Methylocystis* sp. SB2. The signal peptide sequences (shown in Fig S2) were removed from the original MbnT2 and MbnT sequences of *M. trichosporium* OB3b and *Methylocystis* sp. SB2, respectively. White letters on a black background indicate amino acids that are identical in the sequences. The protein domains of MbnT2-OB3b and MbnT-SB2 were searched and predicted using Pfam database. Reference: Notredame C, Higgins DG, Heringa J. 2000. T-Coffee: A novel method for fast and accurate multiple sequence alignment. J Mol Biol 302:205-217.
