# Supplementary material for: Multiple Mechanisms for Copper Uptake by Methylosinus trichosporium OB3b in the Presence of Heterologous Methanobactin
Source: mBio. 2022 Sep 21;13(5):e02239-22. doi: 10.1128/mbio.02239-22 (PMC9601215; doi:10.1128/mbio.02239-22)
Supplement: FIG S4 [file mbio.02239-22-s0006.docx]

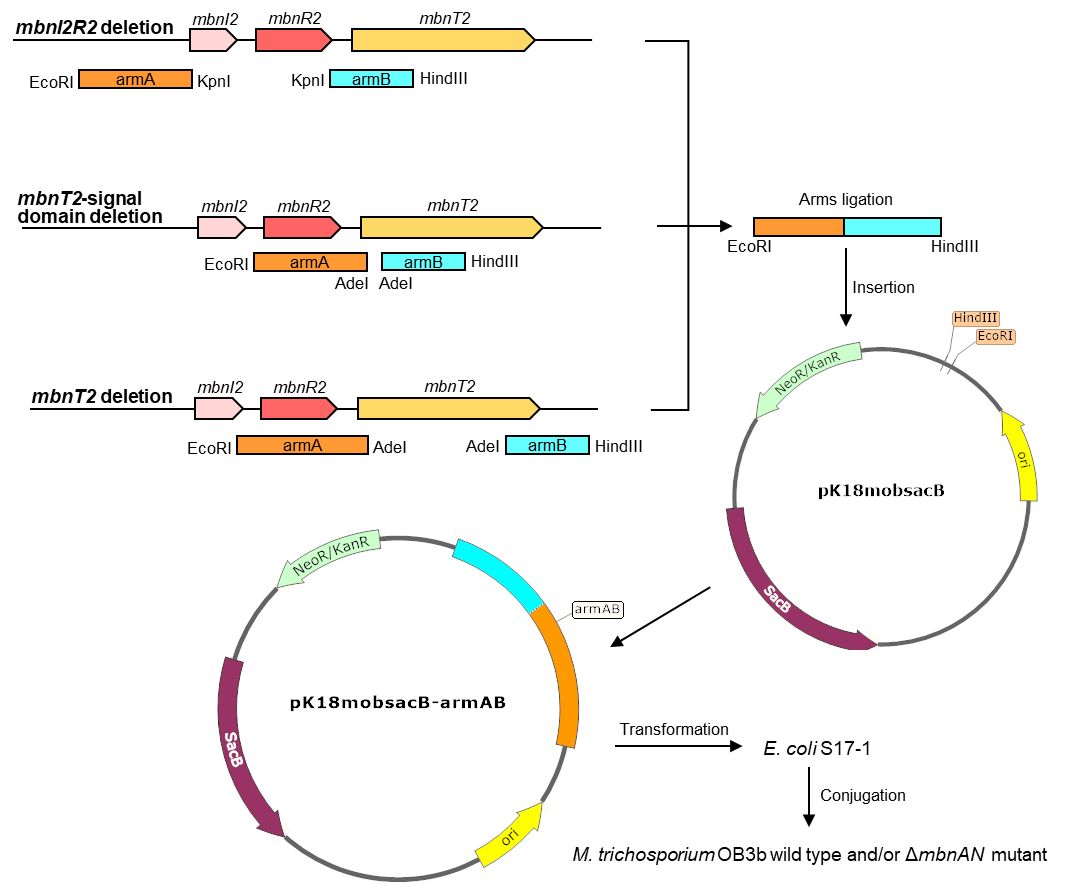


**Fig S4**. Schematic representation of the construction of *M. trichosporium* mutants used in this study.
