# Supplementary material for: Multiple Mechanisms for Copper Uptake by Methylosinus trichosporium OB3b in the Presence of Heterologous Methanobactin
Source: mBio. 2022 Sep 21;13(5):e02239-22. doi: 10.1128/mbio.02239-22 (PMC9601215; doi:10.1128/mbio.02239-22)
Supplement: FIG S5 [file mbio.02239-22-s0007.docx]

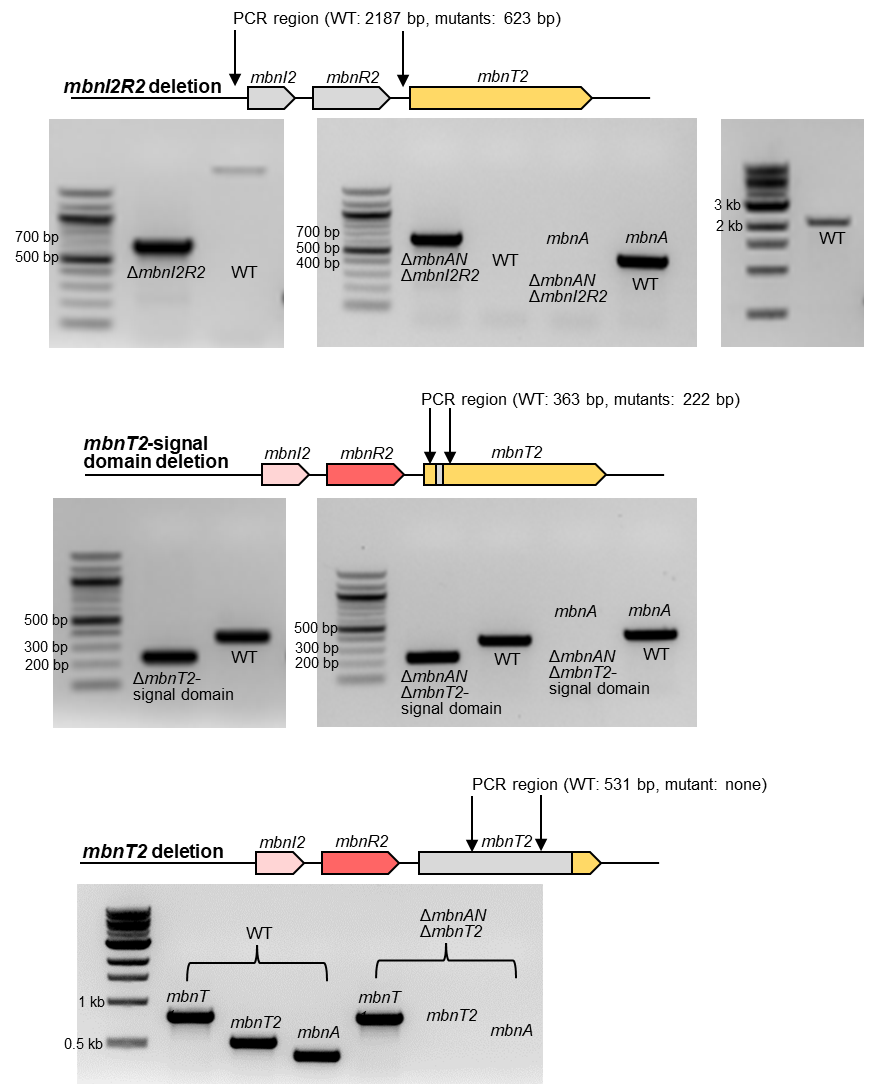


**Fig S5.** Verification of the targeted gene deletion in the constructed *M. trichosporium* mutants by PCR with genomic DNAs extracted from wild type *M. trichosporium* OB3b and the mutants. The PCR regions for verification were indicated. The deletion region of each gene was marked (in grey) in the *mbnT2* gene cluster. *mbnA* gene was also used for verification of the double mutants. *mbnT1* gene was used as a control for verification of the Δ*mbnAN* Δ*mbnT2* mutant.
