# Supplementary material for: Multiple Mechanisms for Copper Uptake by Methylosinus trichosporium OB3b in the Presence of Heterologous Methanobactin
Source: mBio. 2022 Sep 21;13(5):e02239-22. doi: 10.1128/mbio.02239-22 (PMC9601215; doi:10.1128/mbio.02239-22)
Supplement: FIG S6 [file mbio.02239-22-s0008.docx]

**Fig S6.** RT-qPCR analysis of the relative expression of *mbnT* of *Methylocystis* sp. SB2 growing with 1 µM Cu, 1 µM Cu + 5 µM MB-SB2. Error bars indicate standard deviations from triplicate biological cultures. T-test was performed for variance analysis between the growth conditions.
