# Supplementary material for: Multiple Mechanisms for Copper Uptake by Methylosinus trichosporium OB3b in the Presence of Heterologous Methanobactin
Source: mBio. 2022 Sep 21;13(5):e02239-22. doi: 10.1128/mbio.02239-22 (PMC9601215; doi:10.1128/mbio.02239-22)
Supplement: FIG S7 [file mbio.02239-22-s0009.pdf]

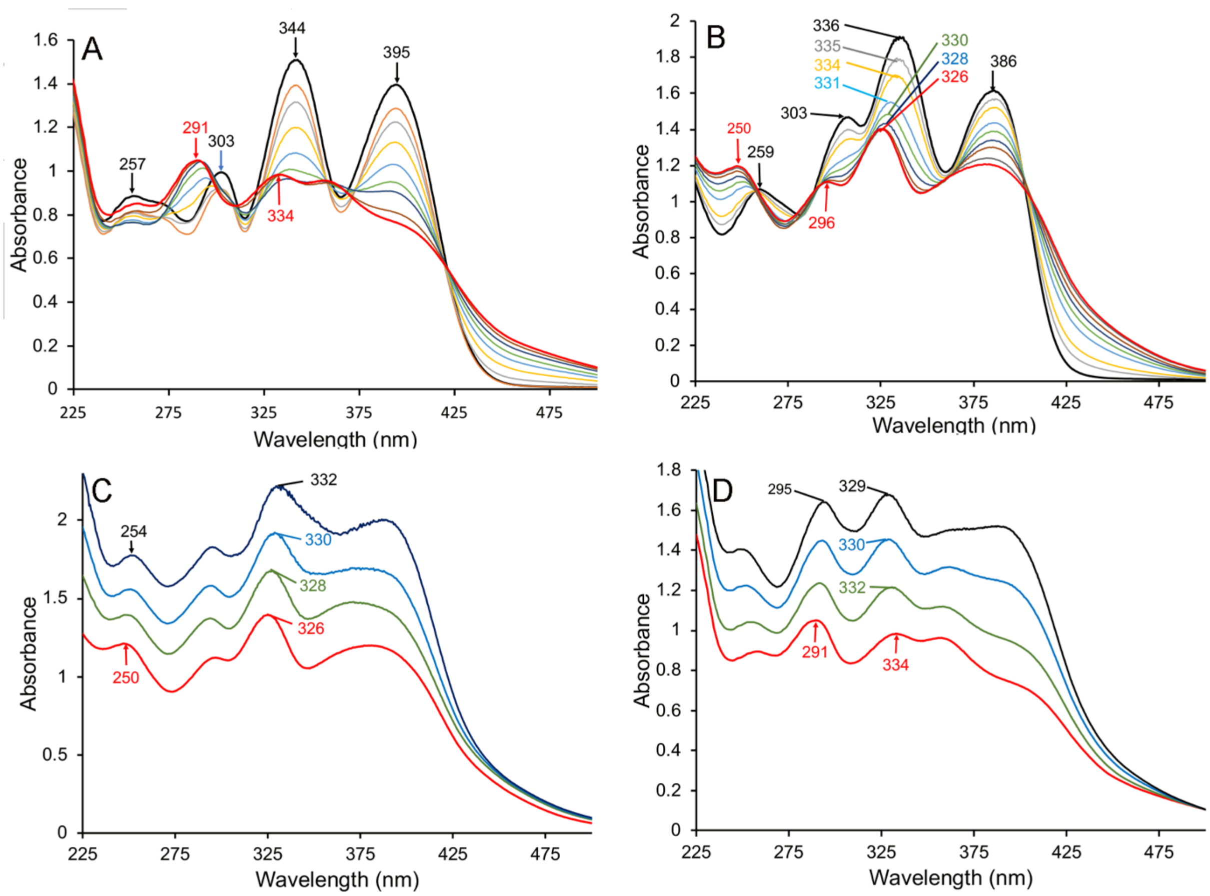

Fig S7. (**A)** UV-visible absorption spectra of 50 nmol ml^{-1} (50 µM) MB-OB3b solution (black line) and following the addition of 5 nmol (orange line), 10 nmol (gray line), 15nmol (yellow line), 20 nmol (light blue line), 25 nmol (green line), 30 nmol (dark blue line), 35 nmol (gold line), or 40 nmol (red line) $CuCl_2$. (**B**) UV-visible absorption spectra of 50 nmol ml^{-1} (50 µM) MB-SB2 solution (black line) and following the addition of 5 nmol (gray line), 10 nmol (orange line), 15 nmol (light blue line), 20 nmol (blue line), 25 nmol (green line), 30 nmol (gold line), 35 nmol (dark blue line), or 40 nmol (red line) $CuCl_2$. (**C**) UV-visible absorption spectra of 50 nmol ml^{-1} (50 µM) MB-SB2 plus 40 nmol $CuCl_2$ (red line) and following the addition of 15 nmol MB-OB3b (green line), 30 nmol MB-OB3b (blue line) or 45 nmol MB-OB3b (black line). (**D**) UV-visible absorption spectra of 50 nmol ml^{-1} (50 µM) MB-OB3b plus 40 nmol $CuCl_2$ (red line) and following the addition of 15 nmol MB-SB2 (green line), 30 nmol MB-SB2 (blue line) or 45 nmol MB-SB2 (black line).
