# Supplementary material for: Multiple Mechanisms for Copper Uptake by Methylosinus trichosporium OB3b in the Presence of Heterologous Methanobactin
Source: mBio. 2022 Sep 21;13(5):e02239-22. doi: 10.1128/mbio.02239-22 (PMC9601215; doi:10.1128/mbio.02239-22)
Supplement: TABLE S1 [file mbio.02239-22-s0001.docx]

Table S1. Potential TBDTs in MB-producing methanotrophs for heterologous and/or homologous MBs uptake

|  | Strain ^a^ | p-MMO | s-MMO | MbnT for homologous MB uptake | | | Potential TBDT/MbnT for heterologous MB uptake | | | | | |
| --- | --- | --- | --- | --- | --- | --- | --- | --- | --- | --- | --- | --- |
|  |  |  |  | Gene locus tag | Signal domain | MbnIR | Gene locus tag ^b^ | Identity (%) ^c^ | Cover-age (%) ^c^ | E-  value ^c^ | Signal domain | MbnIR |
| Group I MB producing methanotrophs | *M. trichosporium* OB3b (NZ_CP023737) ^d^ | + | + | 07180 | + | + | 02135 | 57 | 85 | 0 | + | + |
|  | *Methylosinus sporium* SM89A (NZ_VJMF00000000) | + | + | 19205 | + | + | 12930 | 61 | 98 | 0 | + | + |
|  |  |  |  |  |  |  | 20105 | 44 | 99 | 0 | - | - |
|  | *Methylosinus* sp. 3S-1 (NZ_LXWX01000000) | + | + | 04735 | + | + | 20610 | 57 | 85 | 0 | + | + |
|  | *Methylosinus* sp. LW4 (NZ_ARAB01000000) ^d^ |  |  | 0121405 | + | + | 0111635 | 61 | 87 | 0 | + | + |
|  |  | + | + |  |  |  | 0113945 | 44 | 99 | 0 | - | - |
|  |  |  |  |  |  |  | 0102390 | 41 | 99 | 0 | - | - |
|  | *Methylosinus* sp. PW1 (NZ_JQNK01000000) | + | + | 14665 | + | + | 14740 | 55 | 84 | 0 | + | + |
|  |  |  |  |  |  |  | 22280 | 45 | 99 | 0 | - | - |
| Group II MB producing methanotrophs | *Methylocystis* sp. MitZ-2018 (NZ_PYDU01000000) | + | - | 02775 | - | - | 21200 | 51 | 99 | 0 | + | + |
|  | *Methylocystis rosea* SV97 (NZ_ARCT01000000) ^d^ | + | - | 0101855 | - | - | 0109600 | 35 | 97 | 3×10^-106^ | - | - |
|  | *Methylocystis* sp. SB2 (NZ_CP091318) ^d^ | + | - | 11470 | - | - | 04090 | 35 | 98 | 5×10^-107^ | - | - |
|  | *Methylocystis* sp. B8 (NZ_VBTZ01000000) | + | - | 10000 | - | - | ND ^e^ |  |  |  |  |  |
|  | *Methylocystis* sp. SC2 (NC_018485) | + | - | 06960 | - | - | 12295 | 34 | 97 | 2×10^-116^ | - | - |
|  | *Methylocystis hirsuta* CSC1 (NZ_QWDD01000000) ^d^ | + | + | 17325 | - | - | 13490 | 35 | 97 | 2×10^-112^ | - | - |
|  | *Methylocystis bryophila* S285 (NZ_CP019948) | + | + | 06510 | - | - | 00320 | 50 | 94 | 0 | - | - |
|  |  |  |  |  |  |  | 06510 | 43 | 88 | 0 | - | - |
| Group I and II MB producing methanotrophs | *Methylocystis* sp. LW5 (NZ_JMKQ01000000) | + | + | 0105845 (I) ^f^ | + | + |  |  |  |  |  |  |
|  |  |  |  | 0106300 (II) ^g^ | - | - |  |  |  |  |  |  |
|  | *Methylocystis parvus* OBBP (NZ_CP044331) | + | - | 21355 (I) | + | + |  |  |  |  |  |  |
|  |  |  |  | 13695 (II) | - | - |  |  |  |  |  |  |
|  | *Methylosinus* sp. R-45379 (NZ_LUUM01000000) | + | + | 15795 (I) | + | + |  |  |  |  |  |  |
|  |  |  |  | 15390 (II) | - | - |  |  |  |  |  |  |
|  | *Methylosinus* sp. LW3 (NZ_AZUO01000000) | + | + | 0120540 (I) | + | + |  |  |  |  |  |  |
|  |  |  |  | 0121395 (II) | - | - |  |  |  |  |  |  |
|  | *Methylosinus* sp. sav-2 (NZ_SOEJ01000000) | + | + | 18215 (I) | + | + |  |  |  |  |  |  |
|  |  |  |  | 20070 (II) | - | - |  |  |  |  |  |  |

^a^ Genome accession number for each strain

^b^ MbnT-SB2 and MbnT-OB3b were used as query for searching the potential TBDTs/MbnTs in Group I and II MB producing methanotrophs, respectively

^c^ Comparison between the targeted TBDT and MbnT-SB2 (Group I MB-producing methanotrophs) or MB-OB3b (Group II MB-producing methanotrophs)

^d^ Methanotrophic strain of which MB has been purified and characterized

^e^ ND: Not detected

^f, g^ MbnT for group I (I) and/or group II (II) MB uptake
