# Supplementary material for: Multiple Mechanisms for Copper Uptake by Methylosinus trichosporium OB3b in the Presence of Heterologous Methanobactin
Source: mBio. 2022 Sep 21;13(5):e02239-22. doi: 10.1128/mbio.02239-22 (PMC9601215; doi:10.1128/mbio.02239-22)
Supplement: TABLE S2 [file mbio.02239-22-s0002.docx]

Table S2. Primers used in this study

| **Primer name** | **Sequence (5’―3’)^a^** | **Application** | **Reference** |
| --- | --- | --- | --- |
| qmbnT2_F  qmbnT2_R | GCAATATAGTCCCGGCGTGT AGAAAGGGTATGTCGTGCCG | RT-qPCR | Peng, et al. 2022 |
| qmbnT-SB2_F  qmbnT-SB2_R | AACGTATAGGCCGCGATCAG  ACGTTGAGCAATGGACTCGT | RT-qPCR | This study |
| qmmoX_F  qmmoX_R | TCAACACCGATCTSAACAACG  TCCAGATTCCRCCCCAATCC | RT-qPCR | Knapp, et al., 2007 |
| qpmoA_F  qpmoA_R | TTCTGGGGCTGGACCTAYTTC  CCGACAGCAGCAGGATGATG | RT-qPCR | Knapp, et al., 2007 |
| Eub-341_F  Eub-534_R | CCTACGGGAGGCAGCAG  ATTACCGCGGCTGCTGGC | RT-qPCR | Muyzer et al, 1993 |
| mbnI2R2-armA_F  mbnI2R2-armA_R | ATTTTT gaattc TCTATCGGGAGATCGGCCTT ^b^  ATTTTT ggtacc GTCCGAAATCCCATGAGCCA | Arm PCR | This study |
| mbnI2R2-armB_F  mbnI2R2-armB_R | ATTTTT ggtacc TGATCCTGCTGTACTGACGC ^b^  ATTTTT aagctt AGAAAGGGTATGTCGTGCCG | Arm PCR | This study |
| mbnT2-S-armA_F  mbnT2-S-armA_R | ATTTTT gaattc ACATGAGCAACGGCCAGAAA ^b^  ATTTTT cactttgtg GCTGCGATCGGCGAAGG | Arm PCR | This study |
| mbnT2-S-armB_F  mbnT2-S-armB_R | ATTTTT cacaaagtg TCCGTGTCGATCATTCTCGC ^b^  ATTTTT aagctt AAATGTCGAAATCGCCCAGC | Arm PCR | This study |
| mbnI2R2-check_F ^c^  mbnI2R2-check_R | CCGAGGATCATTGTCTCCAA  ATCGATCCTGCGGGAATATG | PCR for mutant check | This study |
| mbnT2-S-check_F ^c^  mbnT2-S-check_R | GGAACAAGCGTAGAGGCA  CGAGAATGATCGACACGGA | PCR for mutant check | This study |
| mbnT2-check_F ^c^  mbnT2-check-R | CTGAAGACCGTGAATCCGCT  GTCCATTGGCCTGTGTGAGA | PCR for mutant check | Peng et al., 2022 |
| mbnA-check_F  mbnA-check_R | GCGATCAAGTAGGTATAACTTGGAA  CAATTCCTCCCGATCTCTTTC | PCR for mutant check | Gu, et al ., 2017 |
| mbnT1-check_F  mbnT1-check_R | CCGATCGAACCTGGCTCTAT  ATTGTAAATCGTGACGGCGG | PCR for mutant check | Gu, et al., 2016 |

^a^ Y, S, and R are the IUPAC DNA codes for the C/T, C/G, and A/G nucleobases, respectively

^b^ Lowercase letters indicate EcoRI, AdeI, kpnI or HindIII restriction site sequences included in these primers

^c^ Targeting region indicated in Fig S5

References:

Peng P, Kang-Yun CS, Chang J, Gu W, DiSpirito AA, Semrau JD. 2022. Two TonB-dependent transporters in *Methylosinus trichosporium* OB3b are responsible for uptake of different forms of methanobactin and are involved in the canonical ‘copper switch’ Appl Environ Microbiol 88:e01793-21.

Knapp CW, Fowle DA, Kulczycki E, Roberts JA, Graham DW. 2007. Methane monooxygenase gene expression mediated by methanobactin in the presence of mineral copper sources. Proc Natl Acad Sci U S A 104:12040-12045.

Muyzer G, De Waal EC, Uitterlinden AG. 1993. Profiling of complex microbial populations by denaturing gradient gel electrophoresis analysis of polymerase chain reaction-amplified genes coding for 16S rRNA. Appl Environ Microbiol 59:695-700.

Gu W, Baral BS, DiSpirito AA, Semrau JD. 2017. An aminotransferase is responsible for the deamination of the N-terminal leucine and required for formation of oxazolone ring A in methanobactin of *Methylosinus trichosporium* OB3b. Appl Environ Microbiol 83:e02619-16.

Gu W, Haque MFU, Baral BS, Turpin EA, Bandow NL, Kremmer E, Flatley A, Zischka H, DiSpirito AA, Semrau JD. 2016. A TonB-dependent transporter is responsible for methanobactin uptake by *Methylosinus trichosporium* OB3b. Appl Environ Microbiol 82:1917-1923.
